# Exploring cotton plant compounds for novel treatments against brain-eating *Naegleria fowleri*: An *In-silico* approach

**DOI:** 10.1371/journal.pone.0319032

**Published:** 2025-02-24

**Authors:** Aqal Zaman, Sana Noor, Iqra Ahmad, Muhammad Shehroz, Nour Alhajri, Sibtain Ahmed, Umar Nishan, Sheheryar Sheheryar, Riaz Ullah, Abdelaaty A. Shahat, Hanna Dib, Mohibullah Shah

**Affiliations:** 1 Department of Biochemistry, Bahauddin Zakariya University, Multan, Pakistan; 2 Department of Microbiology & Molecular Genetics, Bahauddin Zakariya University, Multan, Pakistan; 3 Department of Bioinformatics, Kohsar University Murree, Murree, Pakistan; 4 College of Engineering and Technology, American University of the Middle East, Kuwait; 5 Hainan International Joint Research Center of Marine Advanced Photoelectric Functional Materials, College of Chemistry and Chemical Engineering, Hainan Normal University, Haikou, Puerto Rico China; 6 Department of Chemistry, Kohat University of Science & Technology, Kohat, Pakistan; 7 Department of Animal Science, Federal University of Ceara, Fortaleza, Brazil; 8 Department of Pharmacognosy, College of Pharmacy, King Saud University Riyadh Saudi Arabia; University of Mashreq, IRAQ

## Abstract

To find potential inhibitors of *Naegleria fowleri* S-adenosyl-L-homocysteine hydrolase (NfSAHH), a brain-eating parasite, structure-based drug design was adopted. *N. fowlerica causes* primary amebic meningoencephalitis (PAM), a fatal central nervous system (CNS) disorder if untreated. NfSAHH protein (PDB ID: 5v96), involved in parasite growth and gene regulation, was targeted and screened against 163 metabolites from *Gossypium hirsutum (cotton* plant). With the aid of different software and web tools, the metabolites were subjected to several analyses. The RMSD was evaluated to validate our molecular docking strategy. Neplanocin A, a common anti-parasitic medication, was used as a reference to select top ligands for post-docking studies. Significant interactions were observed with residues THR-198, HIS-395, and MET-400. The drug-likeness of the top fifty hits was analyzed using Lipinski, Ghose, Veber, Egan, and Muegge rules. The top ten compounds following Lipinski’s RO5 were studied regarding medicinal chemistry, pharmacokinetic simulation, and Swiss target prediction. Advanced strategies, including molecular dynamic simulations, binding energy calculations, and principal component analysis, were employed for the top three hits, namely curcumin, heliocide H2, and piceid, which indicated that heliocide H2 is the most promising candidate, while curcumin and piceid may need further optimization to improve their stability. Overall, the top ten phytochemicals, dotriacontanol, melissic acid, curcumin, 6,6′-dimethoxygossypol, phytosphingosine 2, methyl stearate, stearic acid, piceid, heliocide H2, and 6-methoxygossypol, reported in our study, are worthy enough to be subjected to in vivo and in vitro experimentation to find a novel drug to treat PAM.

## Introduction

*Naegleria fowleri* is an amphizoic amoeba and lives freely in soil and water or as a human parasite [1]. It mostly resides in hotter places (115 ˚F or 46 ˚C), so the spread rate of the parasite amplifies in the summer months, especially during recreational activities like swimming, surfing, water skiing, diving, etc [2]. *Naegleria fowleri* is associated with a serious disease of the central nervous system (CNS) called primary amebic meningoencephalitis (PAM), characterized by meningitis and brain inflammation [1]. Out of the total 30 species of Naegleria, only *Naegleria fowleri* is the reported host of the human body, more common in the youth associated with recreational activities and with domestic water [3]. Religious, leisure, and cultural customs like Ayurveda, ceremonial ablution and cleansing, and the use of neti pots for nasal irrigation have been identified as significant hazards to human health because they can lead to contracting PAM caused by *Naegleria fowleri* [4–6]. This amoeba reaches the brain after adhering to the mucosa of the nose, moving down the olfactory nerves by passing through the cribriform plate. Here it causes severe necrosis and inflammation by consuming the erythrocytes, neurons, and white blood cells [2]. The very first case of a brain-eating parasite, *Naegleria fowleri,* was reported by Fowler and Carter in the year 1965 in South Australia [2]. Later, several cases were reported from the different states of America and the Czech Republic of Europe [7–10]. Because PAM progresses quickly in people and is typically diagnosed postmortem when brain tissue has been stained with hematoxylin and eosin, the disease can be difficult to diagnose [2]. Although PAM survivors are rare, recovery is possible if the illness is identified early and treatment is initiated immediately [11]. To date, amphotericin B in conjunction with rifampin and additional antifungal medications has been the preferred medication for treating PAM [12].

Plants have long been allies of humans in medicine and therapy, with various therapeutic plants being used for the treatment and prevention of diseases since ancient times [13]. Different varieties of medicinal plants have been in use for decades for the treatment of various parasitic infections, i.e., *Cinchona, Azadirachta indica, Acokanthera oppositifolia, Allium cepa, Allium sativum, Cannabis sativa, Datura metel, Mentha spicata, Musa paradisiaca, Nicotiana tabacum, Piper nigrum, Senna italica, Trachyspermum ammi, Trianthema portulacastrum,* and *Vernonia anthelmintica,* etc [14,15]. Similarly, the secondary metabolites, notably, gossypol, of *G. hirsutum* are known to possess various therapeutic properties like anti-cancer, anti-parasitic, anti-viral, anti-fungal, anti-diabetic, anti-inflammatory, and antioxidant, among others [16,17]. Plants like *Rinoreavaundensis* and *Salvia triloba* were reported for the treatment of PAM [18]. Numerous drug targets can be found in the *Naegleria fowleri* proteome, including S-adenosyl-L-homocysteine hydrolase (NfSAHH), phosphoglycerate mutase (NfPGM), protein arginine N-methyltransferase (NfPRMT1), peptidylprolyl isomerase (NfPPI), and prolyl-tRNA synthetase (NfProRS), among others [1,19].

The lack of an FDA-approved medication for treating PAM necessitates the development of new medications to prevent future health emergencies. This study focuses on the NfSAHH protein as a drug target, emphasizing the urgent need for novel drugs to inhibit the proliferation of this deadly disease. NfSAHH catalyzes the breakdown of S-adenosyl-homocysteine (SAH) into adenosine and homocysteine, playing a crucial role in the reproduction and growth of the parasite by facilitating methylation reactions essential for these processes [19]. The research employs phytochemicals from *Gossypium hirsutum* L. (Malvaceae), the cotton plant, by utilizing computer-aided drug design (CADD) to identify potential inhibitors for the NfSAHH protein. The computational/in-silico approaches reduce costs and time and are highly beneficial for the discovery of new medications [20,21]. The secondary metabolites of *G. hirsutum* are known to possess potential anti-parasitic properties, etc [16]. In this study, we aim to validate the potential of these metabolites as inhibitors of NfSAHH through molecular docking, drug-likeness assessments, ADME analysis, molecular dynamics simulations, PK studies, and pharmacophore modeling. Our goal is to discover novel compounds that could offer significant advantages over existing therapies by specifically targeting this critical protein active site and can be developed as the novel NfSAHH protein inhibitor. Further wet lab research is essential to confirm the efficacy of these phytochemicals in inhibiting the target protein.

## Materials and methods

### Ligand preparation

Canonical SMILES for the phytochemicals were retrieved from PubChem and ChemSpider databases. For metabolites without available SMILES, structures were sourced from the literature and manually drawn using ChemDraw Ultra 12.0. A library of 163 secondary metabolites from *G. hirsutum was* prepared in MDL mol format. The database of ligands was prepared, and after the energy minimization process, it was then saved in MDB format.

### Preparation of protein

NfSAHH protein was retrieved from the Protein Data Bank (PDB) under the PDB ID: 5v96 in pdb format. This original protein structure included two co-crystallized ligands, namely adenosine and nicotinamide adenine dinucleotide (NAD). Water molecules were also complex within the protein structure. Water molecules and ligands were deleted to prevent interference with the active site during molecular docking and to create open binding pockets for the desired ligands. It was followed by protonation and energy minimization to ensure the protein has the correct charge state and to optimize its structure by relieving any steric clashes or unfavorable interactions.

### Determination of active site

The site finder tool of Molecular Operating Environment (MOE), 2022.02 Chemical Computing Group ULC, 1010 Sherbrooke St. West, Suite #910, Montreal, QC, Canada, H3A 2R7, MOE2022.v11.18.1) was used to predict the active site, and the largest site was selected [22]. Dummy atoms were created at the selected binding site to define its location and guide the docking process by indicating where potential ligands should bind.

### Molecular docking

The Dock tool of MOE software was used to perform the induced fit docking with the default settings of the software. The docking scores, or S-scores, of the ligands obtained after the docking process determine the capability or tendency of ligands to bind at the active site of the protein.

### Docking validation

MOE software was used to prepare the docked and redocked complexes of the reference compound. Superimposition of the prepared complexes (docked and redocked) was performed in PyMOL (Molecular Graphics System, Version 3.0, Schrödinger, LLC) to obtain the root-mean-square deviation (RMSD) values, on the basis of which the reliability of the docking protocol was evaluated, where values less than 2 Å are considered optimal for proper alignment.

#### Pharmacophore modeling.

The primary pharmacophoric characteristics of the hits were examined to determine the significant pharmacophoric motifs involved in the binding of critical catalytic residues. A high-quality pharmacophore model was developed with the Pharmacophore Query Editor of MOE software. Hydrophobic (Hyd), anionic (Ani), cationic (Cat), aromatic center (Aro), Pi ring center (PiR), aromatic ring or Pi ring normal (PiN), and hydrogen bond donor (Don) were included in the predetermined pharmacophore properties.

### Drug likeness analysis

Drug-likeness analysis of the secondary metabolites was conducted to evaluate their suitability as potential drug candidates based on pharmacokinetic and physicochemical properties. This analysis was done by the SwissADME server, which is a free online web tool [23]. The SMILES of ligands were inserted one by one, and different rules—Lipinski, Ghose, Veber, Egan, and Muegge—were studied to select the top hits.

### Medicinal chemistry

The SwissADME server was used to check the medicinal chemistry of the top hits that adhered to the drug-likeness criteria. PAINS (PAN-Assay Interference Compounds/Frequent Hitters), Brenk, lead-likeness, and synthetic accessibility filters were applied to study the medicinal chemistry of the hits.

### Pharmacokinetics

The pharmacokinetics section of the SwissADME server explains gastrointestinal (GI) absorption, blood-brain barrier (BBB) permeation, P-glycoprotein (P-gp) substrate, inhibition of cytochrome P450 enzymes (CYP1A2, CYP2C19, CYP2C9, CYP2D6, and CYP3A4), and skin permeability coefficient (logKp) of the compounds. These parameters are of great importance in in silico drug design strategies for predicting the pharmacological behavior and potential toxicity of the hits.

### Pharmacokinetic simulation

Pharmacokinetic (PK) modeling was conducted by using PK-Sim software to create and simulate the brain intracellular unbound concentration-time profile of selected metabolites, namely piceid, heliocide H2, and curcumin, in humans to potentially inhibit the target NfSAHH within the amoeba cells in brain tissue. The primary organ selected was the brain, focusing on NfSAHH as a target protein due to its potential role in treating PAM. A virtual population aged 5–15 years old was stimulated, as this age group is most susceptible to this *N. fowleri* infection.

The administration protocol was based on the FAD-approved drug Amphotericin B used for the treatment of *N. fowleri infections.* Intravenous (IV) infusion of a single dose of 1 mg/kg for 2 to 6 hours of infusion time over 24 was set based on the current protocol for Amphotericin B. The brain intracellular compartment, including unbound fractions, was selected as the primary compartment of interest. This selection was based on the intracellular location of the target protein, NfSAHH, within the amoeba cells in the brain tissue. The simulations were performed using a standard model for small molecules in the brain (unbound). For simulation, the physicochemical properties of metabolites, including molecular weight, lipophilicity, plasma protein binding, pKa, and solubility, were taken as input into the model. CYP3A4 was set as the primary metabolizing enzyme for all metabolites [24,25].

### Target fishing analysis

Swiss target prediction, an online web tool, was employed to find out the possible interactions of the top hits with human receptors [26]. The files in.mol format of the metabolites of interest were given as the input, and the predictions were generated. The probability scores were obtained in the range of 0 and 1, and the compounds having a score near 1 or more than 0.5 were more likely to act on the human targets.

### Molecular dynamic simulation

Molecular dynamic (MD) simulations were performed to examine the dynamic interactions between the hits and the protein NfSAHH. These simulations, which lasted 100 ns for each metabolite-protein combination, were conducted using Schroedinger LLC’s Desmond program [27]. By tracking the movements of individual atoms inside the complexes, the simulations, using Newton’s classical motion equations, offered a precise knowledge of the dynamic behavior of the metabolites. Schrödinger’s Maestro was utilized to create the metabolite−NfSAHH complexes for the simulations [28]. To preserve the integrity of the system, this preparation included minimization, optimization procedures, and the inclusion of any missing residues. A 3-point transferable solvent model was used to simulate the solvent environment. It was kept in an orthorhombic simulation box using the force field from OPLS_2005 [29].

Specific parameters were followed during the simulations, including a temperature of 300 K and a pressure of 1 atm. Counterions and 0.15 M sodium chloride were added to the models to mimic physiological conditions and guarantee their neutrality. Every model went through a relaxation phase when the constraints on the system were gradually loosened before the simulations started. For further examination, the paths of these simulations were painstakingly documented. Additional evaluations were conducted on the metabolite−NfSAHH complexes’ stability and behavior. To do this, plots of root-mean-square fluctuation (RMSF) and RMSD were employed.

### Principal component analysis

To perform the essential motions and conformational changes analysis on the molecular dynamics trajectories, principal component analysis (PCA) was used. PCA assists with simplifying the simulation data by focusing on the significant movements of the system, which can provide improved information regarding major structural changes during the interaction. The data for this analysis was projected into the principal components using the inbuilt tools of Schrödinger’s Desmond software to identify the major modes of motion that contribute to the stability and binding of the complexes [30].

### MMGBSA calculations

The binding free energy of the ligand-receptor complexes was calculated using the MMGBSA (Molecular Mechanics Generalized Born Surface Area) approach implemented in Schrödinger’s Prime module [31]. The calculation aimed to evaluate the relative binding affinities and understand the contributions from different energy components. The binding free energy was calculated using the formula:


$$\Delta {\text{G}}_{\text{bind}}={\text{G}}_{\text{complex}}-({\text{G}}_{\text{ligand}}+{\text{G}}_{\text{receptor}})$$

Where, G_complex_, G_ligand_ and G_receptor_ represent the free energies of the complex, the free ligand, and the free receptor, respectively.

**Fig 1:**
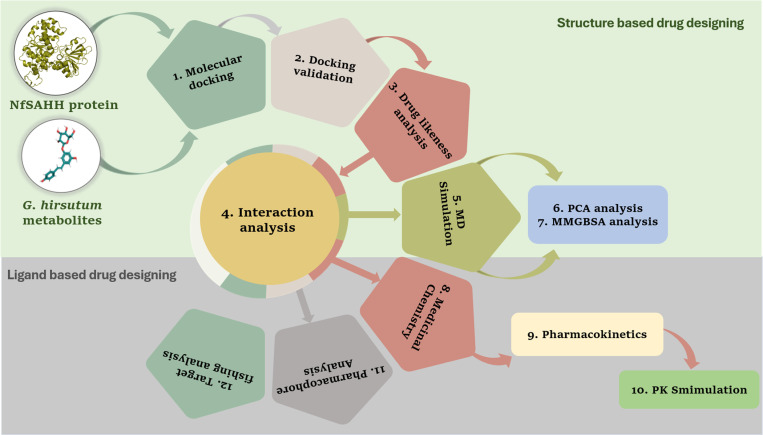
An integrated workflow employing both structure-based and ligand-based drug design was used in this study for the computational design of inhibitory metabolites taken from *Gossypium hirsutum against* the NfSAHH protein.

The comprehensive workflow employed in this study integrates both structure-based and ligand-based drug design approaches to explore potential inhibitory metabolites targeting the NfSAHH protein (Fig 1).

## Results and discussion

### Prediction of active site

**Fig 2:**
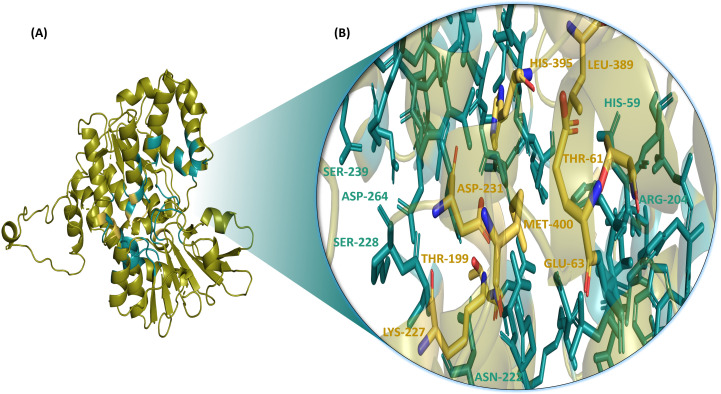
Selected active site of NfSAHH protein (A) with the catalytic residues interacting with the standard compound; Neplanocin A highlighted in yellow (B).

The ligand-binding site of the protein was selected by employing the site finder tool of MOE. The selected active site in the NfSAHH protein was LEU-58, HIS-59, THR-61, GLU-63, THR-64, CYS-83, ASN-84, ILE-85, LEU-111, TYR-114, TRP-115, ASP-136, GLY-137, ASP-139, LEU-142, VAL-163, GLU-165, LEU-166, VAL-169, GLU-197, THR-198, THR-199, THR-200, ARG-204, ASN-222, LYS-227, SER-228, LYS-229, ASP-231, ASN-232, CYS-236, SER-239, CYS-260, GLY-261, PHE-262, GLY-263, ASP-264, VAL-265, GLY-266, LYS-267, THR-283, GLU-284, ILE-285, ASN-289, GLN-292, ALA-316, THR-317, GLY-318, ASN-319, LYS-320, ILE-322, ILE-340, GLY-341, HIS-342, PHE-343, ARG-385, LYS-389, HIS-395, and MET-400 (Fig 2A).

Threonine (THR), glutamic acid (GLU), aspartic acid (ASP), leucine (LEU), and glycine (GLY) residues make up the majority of the active site of NfSAHH. In terms of enzyme activity, these residues are important. For example, because THR, GLU, and ASP can engage in electrostatic interactions and hydrogen bonding, they are frequently implicated in catalytic activity. GLY residues are known for their flexibility, which supports structural changes essential for SAHH enzyme function. Moreover, the Gly-His-Phe sequence in SAHH from Mus musculus and Mycobacterium tuberculosis is conserved across species and contributes to the enzyme’s reaction mechanism [32].LEU may be crucial for the hydrophobic core, preserving its conformation. THR-61, GLU-63, THR-198, LYS-227, ASP-231, LEU-389, HIS-395, and MET-400 are the residues that showed interactions with our standard compound, and our top metabolites also displayed vital binding interactions with these amino acid residues (Fig 2B).

A homo-tetramer is present in the NfSAHH asymmetric unit [33]. Although every chain has an active site, structural study shows that two chains are necessary for the hydrolysis reaction to be successful. Three domains comprise each chain: a C-terminus domain, a substrate-binding domain, and a cofactor-binding domain. The outside of the asymmetric unit, distant from the point where the four subunits meet, is where the substrate-binding domain is found when substrates are not bound. The C-terminus region has a role in protein oligomerization as well as cofactor binding. The structure has two hinge sections that join the substrate-binding and cofactor-binding domains in addition to the three primary components. The hinge region changes shape when substrates bind, sealing the gap between the chain’s cofactor-binding and substrate-binding domains. In the structure of NfSAHH, all subunits exhibit a closed conformation [33].

### Molecular docking

The process of predicting the optimal binding orientation of a ligand to a receptor when the two combine to create a stable complex is known as molecular docking, a type of computer modeling [34]. The library of 163 secondary metabolites of *G. hirsutum* was screened against the NfSAHH protein. 57 metabolites out of 163 exhibited a higher negative docking score than the standard compound, while two remained inactive (S1 Table). The top fifty metabolites having better docking scores than the reference compound Neplanocin A (-7.860 kcal/mol) were selected for the post-docking analysis (S2 Table).

**Fig 3:**
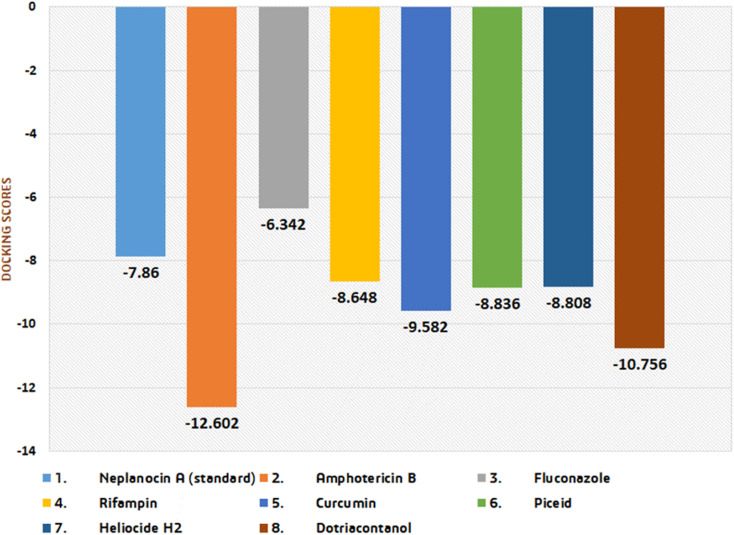
Graphical representation of docking scores of the top compounds of our study: curcumin, piceid, heliocide H2, and dotriacontanol in comparison with the current drugs of choice for the treatment of primary amebic meningoencephalitis (PAM).

Currently, high dosages of amphotericin B are preferred when used with other medications that have been repurposed, such as fluconazole, rifampin, and miltefosine [35,36]. The more negative docking scores of our top metabolites, compared to those of existing medications, including the standard drug, suggest that these metabolites may exhibit greater potency as inhibitors of the target protein (Fig 3). So, our top hits should be subjected to experimental analysis to test their potency, especially the metabolites, namely curcumin, piceid, and heliocide H2.

### Docking validation

**Fig 4:**
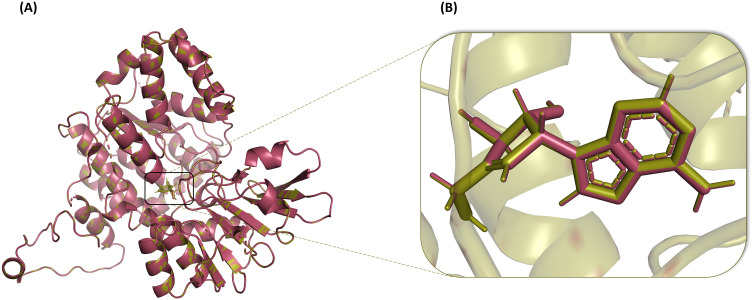
Superimposition of the docked complex (purple) of the standard compound, Neplanocin A, and its redocked complex (green) (A) Zoomed view of the superimposed Neplanocin A from docked (purple) and redocked (green) complexes (B).

The validation of the docking protocol was done to estimate the accuracy of the whole docking procedure. For this purpose, the docked complex and the redocked complex of the reference compound, Neplanocin A, were prepared. These prepared reference complexes were superimposed in the PyMOL software, and the RMSD was calculated and came out to be 0.000 Å, in which the 467 atoms aligned perfectly to 467 atoms (Fig 4A and B). As the RMSD is less than 2 Å approves the reliability of the docking protocol, and the obtained result confirms the perfectness of our docking results.

### Drug likeness analysis

To identify a potential and reliable drug candidate, drug-likeness analysis was conducted using the SwissADME online server on the top fifty metabolites, which were selected based on their docking scores (S-scores) (S2 Table). Lipinski’s rule of 5 (RO5) was applied first to assess the drug-likeness of the metabolites, and the top ten metabolites were selected for further analysis that showed zero or one violation of the RO5. In addition to the number of violations, some other properties, like the number of hydrogen bond donors (less than or equal to 5), the number of hydrogen bond acceptors (less than or equal to 10), the molecular weight (MW; less than or equal to 500 Daltons), and the water-octanol partition coefficient (log P; less than or equal to 5), are critical for determining drug-like characteristics [37]. Three metabolites, namely curcumin, phytosphingosine 2, and heliocide H2, showed zero violations (Table 1), while the seven metabolites, i.e., dotriacontanol, melissic acid, 6,6′-dimethoxygossypol, methyl stearate, stearic acid, piceid, and 6-methoxygossypol, showed one violation for Lipinski’s RO5 (Table 1).

**Table 1:** Drug-likeness analysis of the top ten metabolites and the standard compound following Lipinski, Ghose, Veber, Egan, and Muegge rules and their bioavailability by SwissADME.

Sr. No.	Compound	Lipinski	Ghose	Veber	Egan	Muegge	Bioavailability score
1.	**Neplanocin A** **(standard)**	Yes; 0 violation	No; 1 violation: WLOGP < -0.4	Yes	Yes	Yes	0.55
2.	**Dotriacontanol**	Yes; 1 violation: MLOGP > 4.15	No; 3 violations: WLOGP > 5.6, MR > 130, #atoms > 70	No; 1 violation: Rotors > 10	No; 1 violation: WLOGP > 5.88	No; 3 violations: XLOGP3 > 5, Heteroatoms < 2, Rotors > 15	0.55
3.	**Melissic acid (triacontanoic acid)**	Yes; 1 violation: MLOGP > 4.15	No; 3 violations: WLOGP > 5.6, MR > 130, #atoms > 70	No; 1 violation: Rotors > 10	No; 1 violation: WLOGP > 5.88	No; 2 violations: XLOGP3 > 5, Rotors > 15	0.85
4.	**Curcumin**	Yes; 0 violation	Yes	Yes	Yes	Yes	0.55
5.	**6,6' -dimethoxygossypol**	Yes; 1 violation: MW > 500	No; 4 violations: MW > 480, WLOGP > 5.6, MR > 130, #atoms > 70	Yes	No; 2 violations: WLOGP > 5.88, TPSA > 131.6	No; 1 violation: XLOGP3 > 5	0.55
6.	**Phytosphingosine 2**	Yes; 0 violation	Yes	No; 1 violation: Rotors > 10	Yes	No; 1 violation: Rotors > 15	0.55
7.	**Methyl stearate**	Yes; 1 violation: MLOGP > 4.15	No; 1 violation: WLOGP > 5.6	No; 1 violation: Rotors > 10	No; 1 violation: WLOGP > 5.88	No; 2 violations: XLOGP3 > 5, Rotors > 15	0.55
8.	**Stearic acid (octadecanoic acid)**	Yes; 1 violation: MLOGP > 4.15	No; 1 violation: WLOGP > 5.6	No; 1 violation: Rotors > 10	No; 1 violation: WLOGP > 5.88	No; 2 violations: XLOGP3 > 5, Rotors > 15	0.85
9.	**Piceid**	Yes; 1 violation: NHorOH > 5	Yes	Yes	No; 1 violation: TPSA > 131.6	No; 1 violation: H-don > 5	0.55
10.	**Heliocide H2**	Yes; 0 violation	Yes	Yes	Yes	Yes	0.55
11.	**6-methoxygossypol**	Yes; 1 violation: MW > 500	No; 4 violations: MW > 480, WLOGP > 5.6, MR > 130, #atoms > 70	No; 1 violation: TPSA > 140	No; 2 violations: WLOGP > 5.88, TPSA > 131.6	No; 1 violation: XLOGP3 > 5	0.55

Various other rules were also applied for the analysis of the top ten hits, i.e., Ghose, Veber, Egan, and Muegge. According to Ghose (Amgen), for a compound to be orally active, the value of its log P should be in the range of -0.4 and 5.6; for molar refractivity, this range should be 40 to 130; the number of atoms should be in the range of 20 to 70; MW in between 160 and 480; and the polar surface area (PSA) should be less than 140 [38]. Four metabolites out of the top ten obeyed those rules: curcumin, phytosphingosine 2, piceid, and heliocide H2, while others showed some violations (Table 1). Veber’s rule (GSK filter) states that the number of rotatable bonds of a medicinal compound should be less than 10, the PSA of such compound should be ≤ 140 Å², oral bioavailability ≥ 20%, and the number of H-bonds ≤ 12 [39]. Curcumin, 6,6′-dimethoxygossypol, Piceid, and Heliocide H2 followed Veber’s rule, while the other compounds had one violation each (Table 1). For the Egan rule (Pharmacia filter), the value of log P of the bioactive compound should be less than or equal to 5.88, and the tropological surface area (TPSA) ≥ 131 Å [40]. Curcumin, Phytosphingosine 2, and Heliocide H2 showed no violation of Egan’s rule, and 6,6′-dimethoxygossypol and 6-methoxygossypol showed two violations, while the others showed one violation each (Table 1). Finally, for the Muegge rule (Bayer filter), the MW of such compounds must not exceed 600, lipophilicity should be less than or equal to 7, the number of carbon atoms must be greater than 4, the number of heteroatoms greater than 1, the number of rotatable bonds ≥ 15, the number of H-bond acceptors ≥ 10, and the hydrogen bond donors ≥ 5 [35]. Curcumin and Heliocide H2 followed Muegge’s rules, while the other eight showed some violations (Table 1). The standard compound, Neplanocin A, followed all the rules, including Lipinski, Veber, Egan, and Muegge, with only one exception, i.e., the Ghose rule, with which it showed one violation and has a bioavailability score of 0.55 (Table 1).

### Interactions of ligands with proteins

**Fig 5:**
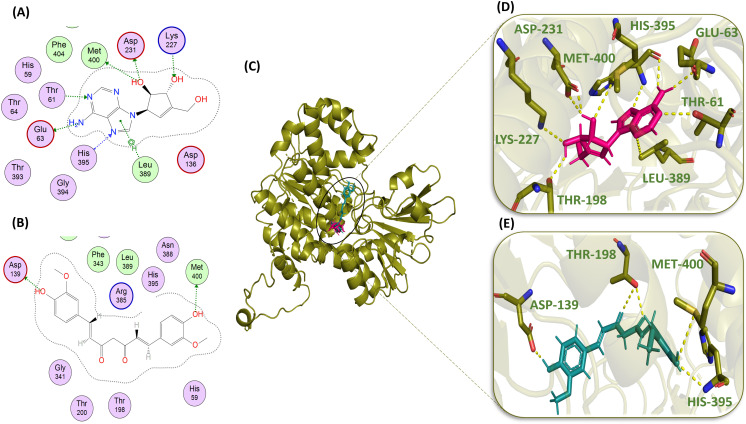
Molecular interaction analysis of standard compound Neplanocin A (pink) and curcumin (blue): 2D schematic representation of Neplanocin A (A) and Curcumin (B) with hydrogen-bonded residue circles; binding of Neplanocin A and Curcumin in the active site of protein (green) (C); 3D interactions of Neplanocin A (D) and Curcumin (E) with active site residues (green) of protein.

The ligand-protein interactions were analyzed by MOE and PyMOL, and 2D and 3D interaction profiles were drawn, respectively. The PyMOL shows only bond distances irrespective of MOE, which also tells about the energies or strengths of the bonds, the interactions observed in the PyMOL software are not explained with the energies in this literature. With the reference compound, Neplanocin A (Fig 5A, C, and D), having the docking score of -7.860, the amino acid residues of the NfSAHH protein showed ten significant interactions; they formed one hydrogen bond with GLU-63 with the bond length of 3.20 Å and the bond energy of -1.8 kcal/mol and two hydrogen bonds with ASP 231; one with the bond length of 2.75 Å and the bond energy of -0.9 kcal/mol and the other at the distance of 2.00 Å. One hydrogen bond with MET-400 with the distance of 3.79 Å and the energy of -0.7 kcal/mol. Two hydrogen bond interactions were exhibited by the amino acid residue HIS-395: one with the bond length and bond energy 2.98 Å and -5.4 kcal/mol, respectively, and the second with the bond distance 2.30 Å. The reference ligand formed one H-bond with THR-61 having the bond length and bond energy 2.93 Å and -1.6 kcal/mol, respectively. One H-bond each for the amino acid residues LYS-227 and LEU-389, with the bond distances and bond energies in the order of 2.95 Å and -3.4 kcal/mol, 3.84 Å and -0.9 kcal/mol, respectively. It also formed a hydrogen bond with THR-198 at the distance of 2.30 Å (Table 2).

**Table 2:** Docking scores, 2D interactions, H-bond distances, and bond energies of the top 10 hits and the standard compound (Neplanocin A).

Sr. No.	Compound	Docking scores	Residues	Interaction	Distance(Å)	Energy(kcal/mol)
1.	**Neplanocin A** **(standard)**	-7.860	GLU-63 ASP-231 ASP-231 MET-400 HIS-395 HIS-395 THR-61 LYS-227 LEU-389 THR-198	H-donor H-donor H-bond H-donor H-acceptor H-bond H-acceptor H-acceptor pi-H H-bond	3.20 2.75 2.00 3.79 2.98 2.30 2.93 2.95 3.84 2.30	-1.8 -0.9 -0.7 -5.4 -1.6 -3.4 -0.9
2.	**Dotriacontanol**	-10.756	ASP-139 ARG-204 ARG-204	H-donor H-bond H-bond	2.99 2.80 2.70	-1.8
3.	**Melissic acid (triacontanoic acid)**	-10.256	ARG-385 ARG-204	H-acceptor H-bond	3.17 3.50	-0.8
4.	**Curcumin**	-9.582	MET-400 ASP-139 THR-198 THR-198 HIS-395	H-donor H-donor H-bond H-bond H-bond	3.65 2.83 2.70 2.80 3.10	-1.7 -3.4
5.	**6,6' -dimethoxygossypol**	-9.416	THR-200 THR-198 ARG-204 ARG-204 THR-200 THR-200	H-acceptor pi-H H-bond H-bond H-bond H-bond	2.89 3.63 2.70 2.90 2.90 2.60	-3.6 -0.6
6.	**Phytosphingosine 2**	-9.123	GLU-165 ILE-85	H-donor H-acceptor	2.96 3.40	-2.3 -1.4
7.	**Methyl stearate**	-8.881	ILE-85 ARG-204	H-acceptor H-bond	3.00 3.10	-1.9
8.	**Stearic acid (octadecanoic acid)**	-8.867	ASN-84 ARG-385	H-bond H-bond	3.30 3.40	
9.	**Piceid**	-8.836	ASP-139 ASP-139 CYS-83 MET-400 PHE-343 HIS-395	H-donor H-donor H-donor H-donor H-acceptor H-bond	3.30 2.74 2.95 3.46 3.26 3.30	-0.8 -1.9 -2.0 -0.9 -1.2
10.	**Heliocide H2**	-8.808	GLU-165 GLU-165 ASN-84 ASP-139 ARG-204 PHE-343	H-donor H-donor H-acceptor H-bond H-bond H-bond	2.72 3.15 2.92 2.70 3.00 3.40	-5.6 -1.9 -2.0
11.	**6-methoxygossypol**	-8.731	GLY-341 THR-200 THR-198 THR-200 ASN-84 ARG-385 GLU-165	H-donor H-acceptor pi-H H-bond H-bond H-bond H-bond	2.91 2.96 3.75 2.80 3.20 3.20 2.60	-1.6 -4.5 -0.8

With one of the top hit metabolites, Dotriacontanol (Fig S1 A, C, and D), the residue ARG-204 formed two hydrogen bonds with the distances of 2.80 Å and 2.70 Å and one hydrogen bond with ASP-139 having the bond distance 2.99 Å and the bond energy of -1.8 kcal/mol (Table 2).

With melissic acid (Fig S1 B, C, and E), ARG-385 formed one hydrogen bond with a bond distance of 3.17 Å and a bond energy of -0.8 kcal/mol; it also formed one hydrogen bond with ARG-204 at a distance of 3.50 Å (Table 2).

In the case of curcumin (Fig 5B, C, and E), MET-400 and ASP-139 formed one H-bond with the bond lengths of 3.65 Å and 2.83 Å and the bond energies of -1.7 kcal/mol and -3.4 kcal/mol, respectively. It also formed two H-bonds with THR-198 with the distances of 2.70 Å and 2.80Å and one with HIS-395 with the bond distance 3.10 Å (Table 2). Interestingly, this interaction profile is similar to the interaction profile of the reference compound, both involving the three residues, i.e., MET-400, THR-198, and HIS-395.

6,6′-dimethoxygossypol (Fig S2 B, C, and E) formed three H-bonds with THR-200 at the distances 2.89 Å, 2.90 Å, and 2.60 Å, with the strength of the first bond being -3.6 kcal/mol. The same metabolite also formed two hydrogen bonds with ARG-204 with the bond lengths 2.70 Å and 2.90 Å and one H-bond with THR-198 (the residue interacting with the standard) with the bond distance and bond energy 3.63 Å and -0.6 kcal/mol, respectively (Table 2).

Phytosphingosine 2 (Fig S3 A, C, and D) formed one hydrogen bond with GLU-165 and one with ILE-85 with the bond distances of 2.96 Å and 3.40 Å and the bond energies of -2.3 kcal/mol and -1.4 kcal/mol, respectively. Another phytochemical, methyl stearate (Fig S3 B, C, and E), formed hydrogen bonds with ILE-85 (3.00 Å, -1.9 kcal/mol) and ARG-204 (3.10 Å). Stearic acid (Fig S2 A, C, and D) formed one H-bond with ASN-84 and one H-bond with ARG-385 with the bond lengths of 3.30 Å and 3.40 Å, respectively (Table 2).

**Fig 6:**
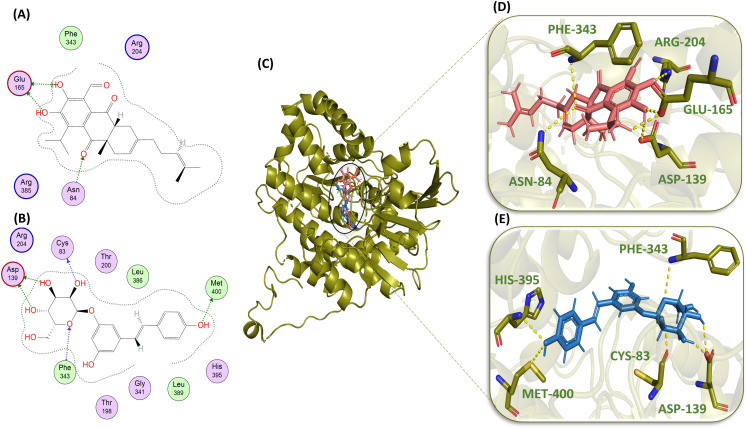
Molecular interaction analysis of standard compound Heliocide H2 (pink) and Piceid (blue): 2D schematic representation of Heliocide H2 (A) and Piceid (B) with hydrogen-bonded residue circles; binding of Heliocide H2 and Piceid in the active site of protein (green); and 3D interactions of Heliocide H2 (D) and Piceid (E) with active site residues (green) of protein.

Piceid (Fig 6B, C, and E) formed six H-bonds: two with ASP-139 with the distances of 3.30 Å and 2.77 Å and the bond energies of -0.8 kcal/mol and -1.9 kcal/mol, respectively; one with CYS-83 (2.95 Å, -2.0 kcal/mol); one with MET 400 (3.46 Å, -0.9 kcal/mol); one with PHE-343 (3.26 Å, -1.2 kcal/mol); and one with HIS-395 (3.30 Å). Again, HIS 395 is the residue interacting with our reference compound too (Table 2). Heliocide H2 (Fig 6A, C, and D) formed two H-bonds with GLU-165 and one H-bond with ASN-84 with the bond lengths of 2.72 Å, 3.15 Å, and 2.92 Å and the bond energies of -5.6 kcal/mol, -1.9 kcal/mol, and -2.0 kcal/mol, respectively, and one H-bond each with the amino acid residues ASP-139, ARG-204, and PHE-343 with the distances of 3.20 Å, 3.20 Å, and 2.60 Å, respectively (Table 2).

6-methoxygossypol (Fig S4 A, B, and C) formed the greatest number of bonds, involving the significant interaction with the THR-198 residue, as it also exhibited a H-bond with our reference compound; seven H-bonds with the amino acid residues, i.e., one H-bond with GLY-341 with the bond distance of 2.91 Å and the bond energy of -1.6 kcal/mol, and two H-bonds with THR-200, one with the bond length of 2.96 Å and the bond energy of -4.5 kcal/mol and the other with the bond length of 2.80 Å. With THR-198, one hydrogen bond was observed with a distance of 3.75 Å, respectively, and a bond energy of -0.8 kcal/mol (Table 2). Other interactions that were observed by this metabolite include the formation of one hydrogen bond with ASN-84 (3.20 Å), one with ARG-385 (3.20 Å), and one with GLU-165 (2.60 Å) (Table 2).

Not only did our top compounds show better binding affinity than the reference compound, but they also exhibited a similar interaction profile. Overall, the significant interactions included those involving the residues MET-400 and HIS-395 with curcumin and peceid, as well as THR-198 with curcumin, 6,6′-dimethoxygossypol, and 6-methoxygossypol. These interactions are noteworthy, as these residues were also part of the interaction profile of the reference compound. Overall, the strong interaction profiles suggest that our top compounds have the potential to be established as novel inhibitors.

### Pharmacophore modeling

**Fig 7:**
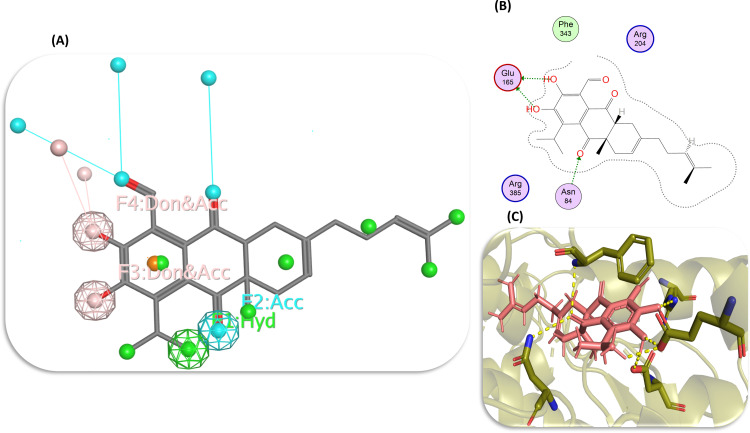
Key pharmacophoric features of Heliocide H2 indicated by F1, F2, F3, and F4 (A), 2D (B) and 3D (C) representations of the binding of the catalytic residues of protein at the important pharmacophoric domains of Heliocide H2.

The approach of ligand-based drug design was performed alongside our structure-based drug design framework to systematically identify the distinctive pharmacophoric motifs of the top metabolites. This technique aimed to optimize their affinity and efficacy in inhibiting the NfSAHH protein, thus realizing their full potential as pharmacological agents. It provides a unique component that may improve selectivity and potency by offering an additional contact point that is uncommon in compounds of a similar kind. It also boosts the capacity of compounds to create hydrogen bonds [41]. Based on their affinity for the essential catalytic residues, the critical pharmacophoric characteristics of the top three hits, i.e., curcumin, piceid, and heliocide H2, were examined. The catalytic residues of the NfSAHH protein were found to interact at the four pharmacophoric scaffolds of the metabolite curcumin identified by F1, F2, F3, and F4. While F1 and F2 indicate hydrogen bond acceptor (Acc), and F3 and F4 exhibit both hydrogen bond acceptor and hydrogen bond donating capability (Don and Acc) (Fig S5 A, B, and C). The addition of rigidifying groups could reduce flexibility and improve its structural stability. Modifications to strengthen hydrogen bond interactions at F3 and F4 may also increase the binding potency of curcumin. The important binding sites of Piceid for the interaction of amino acid residues of the target protein are indicated by F1, F2, F3, F4, and F5 with the Acc descriptor for F1 and the Don and Acc descriptors for the other four pharmacophoric motifs (Fig S5 D, E, and F). Adding nonpolar groups could add diversity of interactions and improve its binding strength. Enhancing the stability of key donor-acceptor features may further improve its biological activity. In the case of heliocide H2, the F1 descriptor indicates Hyd (hydrophobic site), F2 represents Acc, and both F3 and F4 exhibit Don and Acc (Fig 7A, B, and C). The intricate relationship between the molecule and its biological target is explained by emphasizing these pharmacophoric properties, underscoring the significance of each interaction site and spatial arrangement in achieving the intended therapeutic outcome. However, the suggested modifications aim to improve the stability, binding affinity, and biological activity of the top compounds.

### Medicinal chemistry

The medicinal chemistry of the top hit metabolites was studied with the aid of the SwissADME server. The analyses included PAINS, Brenk, lead-likeness, and synthetic accessibility filters. PAINS and Brenk filters actually identify false positive biological outcomes of the compounds [42]. All the top eight hits and the standard compound, Neplanocin A, showed zero PAINS alerts, and the metabolites, Heliocide H2, showed two alerts, and 6-methoxygossypol showed one alert (S3 Table). The Brenk filter showed zero alerts for Dotriacontanol, Melissic Acid, Phytosphingosine 2, Methyl Stearate, and Stearic Acid; one alert for piceid and 6,6′-dimethoxygossypol; and Neplanocin A (standard); two alerts for curcumin and 6-methoxygossypol; and three alerts for Heliocide H2 (S3 Table). The lead-likeness filter indicates the hits that can become leads [43]. None of the hits in our study were predicted to become leads, while our reference ligand, Neplanocin A, can become a lead (S3 Table). The synthetic accessibility score indicates the feasibility of synthesizing a particular compound in wet labs, ranging from 1 to 10. A score near 10 suggests that the compound is too complex to synthesize, while a value near 1 confirms the ease of synthesis of a particular compound [44]. All the top hits of our study and the standard have relatively lower synthetic accessibility scores and are feasible for laboratory synthesis (S3 Table).

### Pharmacokinetics

Absorption, distribution, metabolism, and excretion analysis (ADME analysis) of the top metabolites was done on the SwissADME server. The pharmacokinetics portion of the server describes GI absorption, BBB permeant, P-gp substrate, CYP1A2 inhibitor, CYP2C19 inhibitor, CYP2C9 inhibitor, CYP2D6 inhibitor, CYP3A4 inhibitor, and log*K*_p._ These parameters are focused on examining the availability, absorbance, and response of the metabolites of interest in the body and are extremely important in in silico drug design strategies [45]. The drug must have sufficient solubility to dissolve in GI (gastrointestinal) fluids and pass through the intestinal wall at a speed determined by the intestinal transit. GI absorption of the six metabolites, curcumin, phytosphingosine 2, methyl stearate, stearic acid, piceid, and heliocide H2, was high, while the other four compounds, dotriacintanol, melissic acid, 6,6′-dimethoxygossypol, and 6-methoxygossypol, and the standard compound, Neplanocin A, have relatively low values of GI absorbance (Table 3). None of the metabolites out of the ten top hits and the standard were BBB permeant, but the blood-brain barrier (BBB) permeability could be induced in these metabolites by following various methods of lead optimization, i.e., prodrug strategy [46], lipidization [47], attachment of cell-penetrating peptides [48], and the use of nanocarriers [49] and ultrasound, etc., in order to make a therapeutic medication against CNS infections [50]. P-glycoprotein (P-gp), encoded by the ABCB1 gene, belongs to the ATP-binding cassette of proteins, is very important in pharmacokinetic analysis, and is found in cell membranes of almost all cell types [51]. P-gp (permeability glycoprotein) functions as an efflux pump to transfer material from the intracellular to the extracellular compartment, so a good drug for brain disease must not be the p-gp substrate. Dotriacontanol, melissic acid, phytosphingosine 2, piceid, and Heliocide H2 are P-gp substrates, while curcumin, 6,6′-dimethoxygossypol, methyl stearate, stearic acid, 6-methoxygossypol, and Neplanocin A (standard) are not (Table 3). Inhibition of cytochrome P450 isozymes of the hits was studied by the server. Dotriacontanol, melissic acid, Piceid, and the reference ligand Neplanocin A were found to be the non-inhibitors in every case of these isozymes, while the other metabolites showed different behaviors in each case (Table 3). Methyl stearate and stearic acid are the inhibitors of CYP1A2; 6,6′-dimethoxygossypol, Heliocide H2, and 6-methoxygossypol are the inhibitors of CYP2C19; curcumin and Heliocide H2 are the inhibitors of CYP2C9; phytosphingosine 2 is the inhibitor of CYP2D6; and in the case of CYP3A4, the inhibitors are curcumin and Heliocide H2 (Table 3). The two metabolites, curcumin and piceid, have good Kp values (skin permeability), while the other eight and the standard have relatively lower values of skin permeation (Table 3).

**Table 3:** Pharmacokinetics of the top 10 metabolites and the standard studied by SwissADME.

Sr. No.	Compound	GI absorption	BBB permeant	P-gp substrate	CYP1A2 inhibitor	CYP2C19 inhibitor	CYP2C9 inhibitor	CYP2D6 inhibitor	CYP3A4 inhibitor	Log *K*_p_ (skin permeation)
1.	**Neplanocin A (standard)**	Low	No	No	No	No	No	No	No	-9.31 cm/s
2.	**Dotriacontanol**	Low	No	Yes	No	No	No	No	No	2.06 cm/s
3.	**Melissic acid (triacontanoic acid)**	Low	No	Yes	No	No	No	No	No	1.38 cm/s
4.	**Curcumin**	High	No	No	No	No	Yes	No	Yes	-6.28 cm/s
5.	**6,6' -dimethoxygossypol**	Low	No	No	No	Yes	No	No	No	-4.25 cm/s
6.	**Phytosphingosine 2**	High	No	Yes	No	No	No	Yes	No	-4.94 cm/s
7.	**Methyl stearate**	High	No	No	Yes	No	No	No	No	-2.19 cm/s
8.	**Stearic acid (octadecanoic acid)**	High	No	No	Yes	No	No	No	No	-2.19 cm/s
9.	**Piceid**	High	No	Yes	No	No	No	No	No	-7.95 cm/s
10.	**Heliocide H2**	High	No	Yes	No	Yes	Yes	No	Yes	-5.33 cm/s
11.	**6-methoxygossypol**	Low	No	No	No	Yes	No	No	No	-4.40 cm/s

Overall, the ADME analysis provided significant information about the pharmacokinetics of the top metabolites, highlighting their GI absorption, P-gp substrate status, cytochrome P450 inhibition, and skin permeability. Notably, curcumin and piceid exhibited high GI absorption and favorable skin permeability, while none of the top metabolites were BBB permeant, necessitating further lead optimization for CNS-targeted applications. The identification of P-gp substrates and non-substrates, as well as inhibitors of specific cytochrome P450 isozymes, highlights the complexity of optimizing these metabolites for therapeutic use, with curcumin and heliocide H2 showing diverse inhibition profiles that could influence their pharmacokinetic properties. Further experimental studies are needed to confirm these results.

### Concentration-time profiles of selected metabolites

The concentration time profile for the unbound fraction of three selected metabolites, namely piceid, heliocide H2, and curcumin, was generated through physiologically-based pharmacokinetic (PBPK) simulation in the brain intracellular matrix of fluid over a 24-hour period of time. These profiles indicated their potential effectiveness and duration of action in the target tissue.

**Fig 8:**
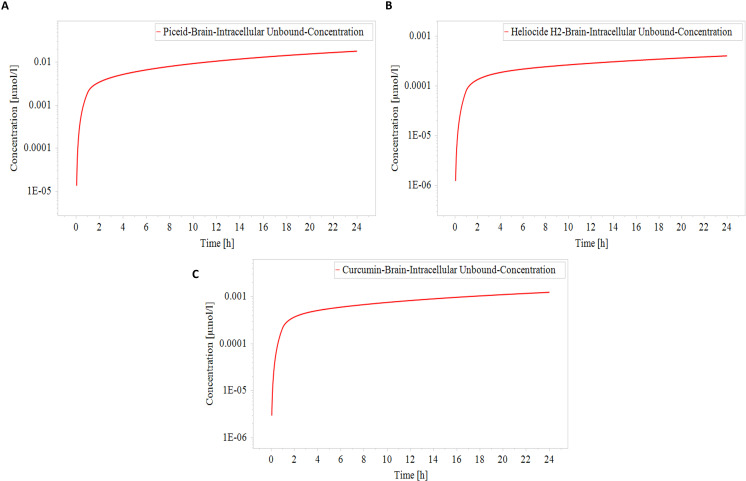
Concentration time profile of metabolites shown in (A) Piceid, (B) Heliocide H2, and (C) Curcumin. The red solid line represents varying concentrations of these metabolites in the brain’s intracellular matrix of fluid over a 24-hour period of time.

Piceid showed the most promising pharmacokinetic profile among the three metabolites. Its brain intracellular unbound concentration rapidly peaked at approximately 0.01 μmol/L within the first few hours post-administration (Fig 8A). Remarkably, it maintained this concentration throughout the 24-hour period. This sustained high concentration suggests excellent potential for once-daily dosing. Heliocide H2 showed a similar profile to Piceid but at a considerably lower level. Its unbound concentration in the brain intracellular compartment started initially at 0.0003 μmol/L and remained below 0.001 μmol/L within a 24-hour period (Fig 8B). The sustained low concentration of heliocide exhibits a low potential efficacy compared to Piceid against *N. fowler, suggesting* a more frequent dosing. Curcumin exhibited an intermediate concentration profile, reaching above 0.001 μmol/L (Fig 8C). It maintained a relatively stable concentration throughout the 24-hour period. This steady-state concentration demonstrates high potency against the target.

Comparatively, the peak concentrations and constant levels can be classified as piceid>  heliocide H2>  curcumin. The higher and more sustained brain intracellular unbound concentrations of Piceid suggest the most promising pharmacokinetic profile for targeting *N. fowleri* in the brain. Its moderate lipophilicity value and high unbound fraction in plasma contribute to its superior brain penetration and retention time. For therapeutic concentration in the brain intracellular compartment, turmeric may require higher dosing to improve its pharmacokinetic profile for potential use as an anti-amoebic agent. Therefore, all three metabolites show the ability to cross the blood-brain barrier and maintain relatively stable concentrations in the brain intracellular compartment. However, the concentrations vary significantly between metabolites, which could be significant for suggesting their potential efficacy against *N. fowleri*. It must be noted that the BBB permeability results from the SwissADME webserver are contradictory to these results, where it did not predict the BBB permeable nature of these three metabolites. The difference in these results might be due to the variable algorithms that form the bases of these two different software. The PBPK simulations dynamically model drug distribution in tissues and organs and predict the behavior of compounds in a physiological system, while SwissADME predictions rely on static cheminformatics algorithms based on molecular properties. PBPK results provide a more detailed and realistic assessment [23,52,53]. Experimental studies are needed to confirm the permeability of these metabolites. The efficacy of these metabolites would ultimately depend on their binding affinity and inhibitory potency against the target protein, NfSAHH, which requires in vitro and in vivo studies.

### Swiss target prediction

**Fig 9:**
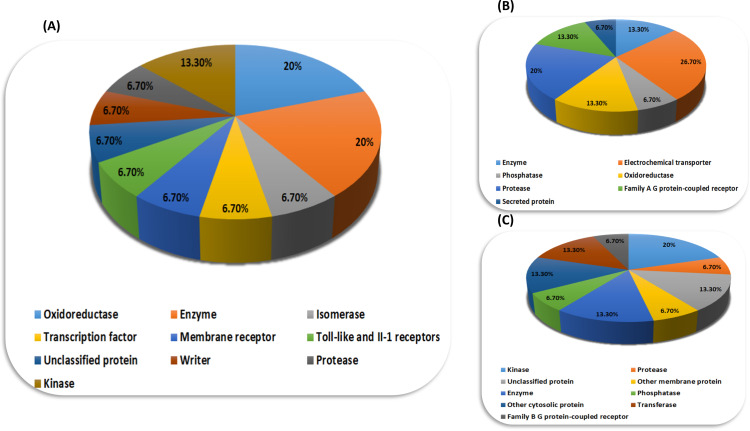
Pie charts of target prediction of the top hits, i.e., (A) cucumber, (B) pineapple, and (C) heliocide H2.

Target fishing analysis was done to evaluate the potency of the top hits and their adverse effects on the human body. It is possible that the compounds that are involved in the inhibition of the parasitic protein (NfSAHH) can also interfere with the normal metabolic processes occurring in the human body by deteriorating the structure or function of the human proteins [54]. This analysis can be so promising in the case of drug repurposing, as it is possible that the compounds showing adverse effects in one case could have some other therapeutic properties [55]. The probability score tells us about the likeness of the test compound to be the protein target, and the score less than 0.5 indicates the safety and reliability of the trial compound to be used by human beings [54]. Interestingly, the standard compound, Neplanocin A, has not a single predicted target, which makes it a good drug of choice. Six top hits, Dotriacontanol, Melissic Acid, Phytosphingosine 2, Methyl Stearate, Piceid (Fig 9B), and Heliocide H2 (Fig 9C), showed excellent results with probability scores less than 0.5 and are predicted to be safe for the human body (S4 Table). The probability score of 6-methoxygossypol was exactly 0.51, and that of 6,6′-dimethoxygossypol was 0.66. Curcumin (Fig 9A) and stearic acid showed extremely high probabilities of interacting with the human proteins, i.e., 100% and 92%, respectively (S4 Table). Monoamine oxidase A, beta amyloid A4 protein, histone acetyltransferase p300, prostaglandin E synthase, and toll-like receptor (TLR7/TLR9) were among the predicted targets of curcumin, and for stearic acid, some were peroxisome proliferator-activated receptor alpha, peroxisome proliferator-activated receptor delta, fatty acid-binding protein adipocyte, fatty acid-binding protein epidermal, and fatty acid-binding protein muscle.

### Molecular dynamics simulation

**Fig 10:**
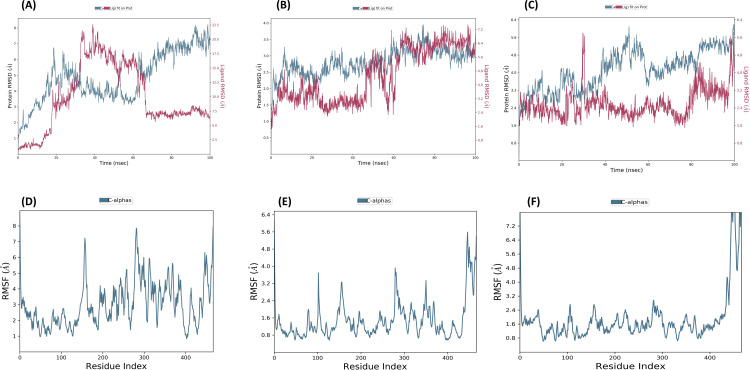
RMSD and RMSF plots for top metabolites and NfSAHH complexes. (A) RMSD plot for the Curcumin-NfSAHH complex; (B) RMSD plot for the Heliocide H2-NfSAHH complex; (C) RMSD plot for the Piceid-NfSAHH complex (D). Protein RMSD is shown in blue, and ligand RMSD is shown in red. RMSF plot for the Curcumin-NfSAHH complex; (E) RMSF plot for the Heliocide H2-NfSAHH complex; (F) RMSF plot for the Piceid-NfSAHH complex.

**Fig 11:**
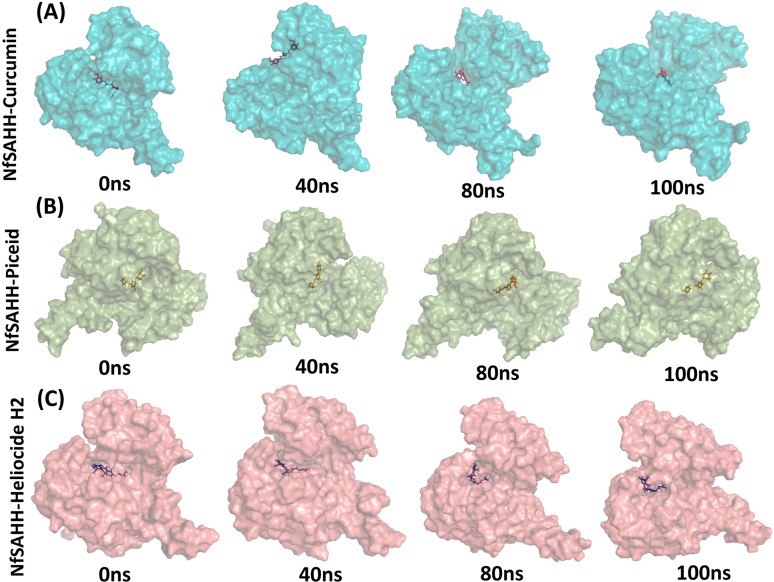
MD snapshots of ligand-NfSAHH complexes at 0 ns, 40 ns, 80 ns, and 100 ns. (A) Curcumin-NfSAHH complex, (B) Piceid-NfSAHH complex, and (C) Heliocide H2-NfSAHH complex. The snapshots illustrate the positional behavior of the ligands over the 100 ns simulation timeline.

The Desmond program from Schrodinger LLC was used for simulations of molecular dynamics. For each of the three top complexes —curcumin-NfSAHH, heliocide-H2-NfSAHH, and piceid-NfSAHH —the simulation took 100 ns. Prior to using the atom selection to compute the RMSD, each protein frame is aligned using the reference frame backbone. The RMSD in the bound and unbound states of the metabolites and proteins was computed and presented as a histogram against the Ca atoms of the protein in order to interpret the conformational stability and dynamic features from the initial configuration to the final state (Fig 10). The docked complex is stable when there are slight deviations from the RMSD curve and vice versa. In the case where NfSAHH is in complex with curcumin, heliocide H2, and piceid, this is the scenario. The computed RMSD for the Curcumin NfSAHH complex was 8.0 ±  1 Å, exhibiting abrupt fluctuations at 20, 35, and 60 ns (Fig 10A). The computed RMSD for the Heliocide H2 and Piceid complexes with NfSAHH were 3.50 ±  1 and 5.80 ±  1 Å, in that order (Fig 10B and C). Of the three, the heliocide H2NfSAHHRMSD had the highest degree of stability, with only a small fluctuation at 60 ns, while the Piceid NfSAHH complex exhibited fluctuations at 30 and 80 ns. Overall, the RMSD plots showed that the heliocide H2 is the most promising candidate, while curcumin and piceid may need further optimization to improve their stability. The greater reported fluctuations in RMSD of complexes may be explained by the presence of naturally flexible regions. This observable fluctuation pattern is consistent with the findings of previous studies and lends credence to the theory of structural dynamics in this context [56]. The stability of the metabolites in relation to NfSAHH and its binding pocket is represented by the ligand RMSD. If the readings are significantly higher than the RMSD of protein, it is likely that the ligand has diffused away from its initial binding site. The NfSAHH complexes and all three metabolites showed lower ligand values than the protein. MD snapshots (Fig 11) clearly show that while the other two showed positional adjustments, Heliocide H2 stayed within the binding pocket over the entire 100 ns simulation.

Using RMSF, the local changes along the protein chain can be described. Peaks in the RMSF plots represent the areas of the protein that fluctuate the greatest during the simulations. In order to gain a better understanding of the stability of the complexes that were created, we conducted a residual flexibility study. The results of this analysis match our findings, showing that all three metabolites and NfSAHH complexes have lower values of flexibility (Fig 10D, E, and F).

### Principal component analysis

**Fig 12:**
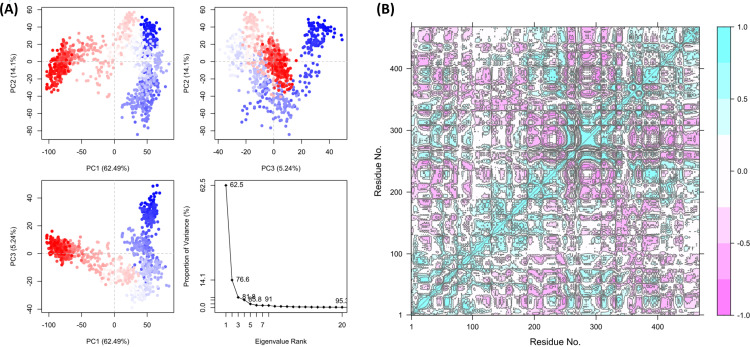
PCA analysis of protein-ligand complexes for curcumin (A) PCA scatter plots demonstrate the distribution of conformational states for curcumin. The clustering indicates distinct conformational shifts, with a gradient from red to blue representing progression over the trajectory along with the scree plot, highlighting significant conformational changes. (B) The residue cross-correlation matrix reveals correlated (cyan) and anti-correlated (pink) motions, suggesting large-scale changes in the protein structure affecting functional sites.

The PCA analysis of the protein-ligand complexes for Curcumin, Heliocide H2, and Piceid provides valuable insights into the dynamic behavior and conformational changes induced by each ligand. Starting with curcumin, the PCA scatter plots (Fig 12A) reveal the distribution of conformational states along the principal components. PC1 explains the majority of the variance (62.49%), followed by PC2 (14.1%) and PC3 (5.24%). The clustering in the scatter plots (PC1 vs. PC2, PC1 vs. PC3, and PC2 vs. PC3) indicates distinct conformational shifts, with a gradient from red to blue suggesting progression in sampled states over the trajectory. The scree plot (Fig 12A) demonstrates a sharp decline in variance explained after PC1, emphasizing that the most significant conformational changes are captured predominantly by this component. This suggests that curcumin induces major changes primarily along a single dominant mode. The residue cross-correlation matrix (Fig 12B) for curcumin reveals patterns of correlated and anti-correlated motions across residues. Positive correlations (cyan) and negative correlations (pink) indicate regions of concerted or opposing movements, suggesting that the protein undergoes large-scale changes, possibly affecting functional sites.

**Fig 13:**
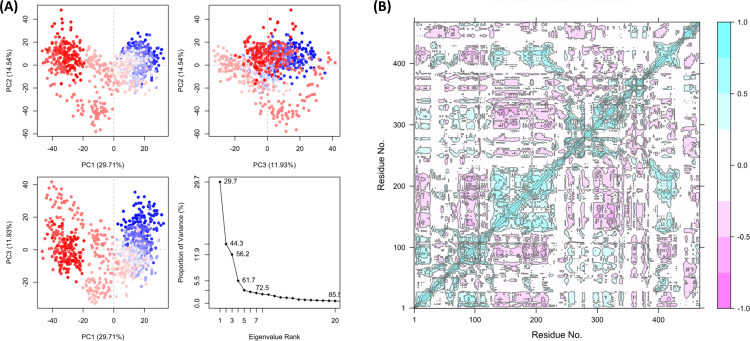
PCA analysis of protein-ligand complexes for Heliocide H2 (A) PCA scatter plots demonstrate the distribution of conformational states for Curcumin. The clustering indicates distinct conformational shifts, with a gradient from red to blue representing progression over the trajectory along with the scree plot, highlighting significant conformational changes. (B) The residue cross-correlation matrix reveals correlated (cyan) and anti-correlated (pink) motions, suggesting large-scale changes in the protein structure affecting functional sites.

For Heliocide H2, the PCA scatter plots (Fig 13A) indicate a more complex distribution of conformational states compared to Curcumin. PC1 explains 29.71% of the variance, with PC2 and PC3 explaining 14.54% and 11.93%, respectively. The reduced variance explained by PC1 compared to curcumin suggests that the conformational changes are spread across multiple components, implying a broader range of dynamic behaviors. The scatter plots exhibit less distinct clustering, indicating greater variability or fewer distinct states. The scree plot (Fig 13A) shows a more gradual decline, supporting the idea that multiple components contribute to the overall dynamics. The residue cross-correlation matrix (Fig 13B) reveals diffuse patterns of correlated and anti-correlated motions, indicating that heliocide H2 binding results in different dynamic interactions across the protein. The overall pattern suggests a more distributed influence on the protein’s motion, possibly reflecting different binding characteristics compared to curcumin.

**Fig 14:**
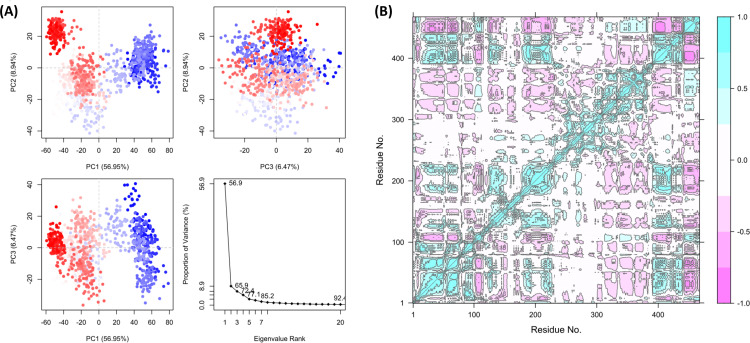
PCA analysis of protein-ligand complexes for Piceid (A) PCA scatter plots demonstrate the distribution of conformational states for curcumin. The clustering indicates distinct conformational shifts, with a gradient from red to blue representing progression over the trajectory along with the scree plot, highlighting significant conformational changes. (B) The residue cross-correlation matrix reveals correlated (cyan) and anti-correlated (pink) motions, suggesting large-scale changes in the protein structure affecting functional sites.

For Piceid, the PCA scatter plots (Fig 14A) show that PC1 explains 56.95% of the variance, followed by PC2 (8.94%) and PC3 (6.47%). Similar to curcumin, PC1 captures a substantial portion of the conformational variability, although not as high. The clustering observed in PC1 vs. PC2 and PC1 vs. PC3 plots suggests that significant structural changes are driven by PC1, with red and blue colors indicating distinct clusters of conformations sampled during the simulation. The scree plot (Fig 14A) indicates that PC1 is the dominant mode of motion, with subsequent components contributing less to the overall variance. The residue cross-correlation matrix (Fig 14B) shows distinct patterns of correlated and anti-correlated motions. Unlike the other two ligands, the regions of correlation are more distributed, suggesting that Piceid affects the protein’s dynamics differently. The diffused cross-correlation pattern may imply a less rigid binding interaction, leading to distributed changes across the protein.

**Fig 15:**
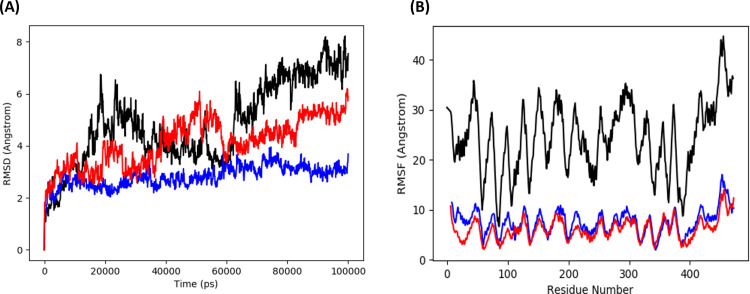
Combined plots of (A) RMSD and (B) RMSF for Curcumin (black), Heliocide H2 (blue), and Piceid (red), derived from the trajectory data obtained through Principal Component Analysis (PCA).

The combined RMSD and RMSF plots (Fig 15A and B) provide further perspective about the dynamic stability and flexibility induced by each ligand. The RMSD plot (Fig 15A) reveals that curcumin (black line) induces the highest deviation, reaching values of around 8 Å by the end of the simulation. This indicates significant conformational changes, suggesting that curcumin binding may not stabilize the protein as effectively. In contrast, Heliocide H2 (blue line) maintains a consistently lower RMSD, reaching a maximum of about 3-4 Å, implying the highest structural stability and minimal deviation. Piceid (red line) exhibits intermediate RMSD, stabilizing around 4-6 Å, suggesting moderate stability compared to the other two ligands. The RMSF plot (Fig 15B) shows that curcumin (black line) induces high local flexibility, with RMSF values reaching up to 40 Å for certain residues. This implies that curcumin binding causes considerable fluctuations, particularly in loop or terminal regions. Heliocide H2 (blue line) exhibits the lowest RMSF values, staying below 10 Å, which indicates minimal residue-level flexibility and suggests that the protein maintains a stable conformation. Piceid (red line) shows moderate flexibility, with RMSF values around 10-15 Å, which is consistent with the RMSD results and suggests moderate influence on the protein’s dynamics.

Overall, the PCA, RMSD, and RMSF analyses collectively highlight the different impacts of each ligand on the protein’s dynamics. Curcumin induces the most significant conformational changes and flexibility, as evident from the high variance explained by PC1, the RMSD, and the RMSF plots. Heliocide H2, in contrast, results in the most stable protein-ligand interaction, with low RMSD and RMSF values and a more distributed influence across multiple principal components. Piceid shows intermediate behavior, affecting the protein dynamics to a moderate extent. These results are consistent with the MMGBSA findings, where heliocide H2 exhibited more favorable binding stability compared to curcumin and piceid. The differences in correlated and anti-correlated motions observed in the cross-correlation matrices also provide clarification into how each ligand differentially modulates the protein’s internal dynamics, potentially leading to different functional implications.

### MMGBSA analysis

The MMGBSA analysis results show the binding free energy for curcumin, heliocide H2, and piceid at two different time points (0 ns and 100 ns). For all three ligands, the binding free energy (ΔG_bind) becomes less negative at 100 ns compared to 0 ns, suggesting a decrease in binding affinity over time. This trend indicates potential conformational changes in either the ligand or receptor, leading to a weakened interaction. Initially, curcumin has the most favorable binding affinity at -92.52 kcal/mol, closely followed by heliocide H2 (-90.78 kcal/mol), with piceid having the least favorable binding affinity at -79.89 kcal/mol. By 100 ns, the binding energy decreases for all ligands, with curcumin at -69.70 kcal/mol, heliocide H2 at -63.35 kcal/mol, and piceid at -58.44 kcal/mol. Curcumin maintains the highest binding energy at both time points, indicating stronger binding compared to the other ligands (Table 4).

**Table 4:** Comparison of MMGBSA Binding Free Energy Components for Curcumin, Heliocide H2, and Piceid at 0 ns and 100 ns (The energy components include Coulombic interactions, covalent interactions, hydrogen bonding (Hbond), lipophilic (hydrophobic) interactions (Lipo), packing interactions, solvation energy computed by the Generalized Born (Solv_GB) model, and van der Waals (vdW) interactions).

Compound	Curcumin		Heliocide H2		Piceid	
**Time**	**0ns**	**100ns**	**0ns**	**100ns**	**0ns**	**100ns**
r_psp_MMGBSA_dG_Bind	-92.5168	-69.7008	-90.776	-63.348	-79.8945	-58.4386
r_psp_MMGBSA_dG_Bind_Coulomb	-30.1749	-25.1628	-19.7654	-8.84613	-49.0991	-47.3936
r_psp_MMGBSA_dG_Bind_Covalent	11.59609	1.813067	5.27556	-0.09729	7.474058	29.50948
r_psp_MMGBSA_dG_Bind_Hbond	-2.3696	-0.80147	-0.89159	-1.1179	-1.97346	-1.67084
r_psp_MMGBSA_dG_Bind_Lipo	-43.0909	-26.2616	-51.1873	-30.32	-38.4869	-37.6991
r_psp_MMGBSA_dG_Bind_Packing	-1.98233	-2.73234	-1.41031	-0.09534	-0.13295	-1.31547
r_psp_MMGBSA_dG_Bind_SelfCont	0	0	0	0	0	0
r_psp_MMGBSA_dG_Bind_Solv_GB	33.1771	26.21049	39.86021	21.98703	50.51056	46.46999
r_psp_MMGBSA_dG_Bind_vdW	-59.6723	-42.7661	-62.6572	-44.8584	-48.1868	-46.339

The change in individual interaction contributions provides an explanation for the decrease in binding affinity. Coulombic interactions become less negative at 100 ns for all ligands, indicating weaker electrostatic interactions over time. For curcumin, the Coulomb contribution decreases from -30.17 at 0 ns to -25.16 at 100 ns, and a similar trend is observed for piceid, which changes from -49.10 to -47.39. This reduction suggests possible structural rearrangements that weaken electrostatic contacts. Lipophilic contributions also decrease for all ligands over time, indicating reduced hydrophobic interactions. For curcumin, this change is significant, from -43.09 to -26.26, suggesting that the ligand may be moving away from hydrophobic regions in the receptor binding site. van der Waals interactions, which contribute to the stabilization of the complex, also decrease in magnitude over time. For curcumin, this contribution changes from -59.67 at 0 ns to -42.77 at 100 ns. Heliocide H2 and Piceid show similar decreases in van der Waals interactions, implying that the contacts between the ligand and receptor are weakening as time progresses (Table 4). Hydrogen bonding contributions also decrease for all ligands, though the magnitude of this decrease is smaller compared to Coulombic, lipophilic, and van der Waals interactions. For curcumin, the hydrogen bonding energy changes from -2.37 to -0.80, indicating fewer effective hydrogen bonds are formed over time. This reduction in favorable interactions across all components points to a less stable ligand-receptor complex at 100 ns (Table 4).

While the MMGBSA analysis showed a decrease in binding energy over time, this is likely due to conformational flexibility and equilibrium dynamics rather than a real loss of binding [57,58]. Importantly, all compounds still maintained negative binding energies, which means their interactions remain favorable (e.g., Heliocide H2: -63.348 kcal/mol). Overall, the decrease in binding affinity for all three ligands over time can be attributed to conformational changes that reduce the strength of Coulombic, hydrophobic, and van der Waals interactions. Among the three ligands, Heliocide H2 shows strong initial binding, indicating it may also be a viable candidate for further study. To improve binding affinity and maintain stability over time, modifying functional groups to enhance electrostatic, hydrophobic, or hydrogen bonding interactions may be beneficial. Further molecular dynamics simulations could provide a deeper understanding of the structural changes responsible for decreased binding affinity and guide the design of more stable ligand analogs.

## Conclusions

*Naegleria fowleria causes* a serious disorder of the central nervous system in human beings, PAM, and the deadly disease needs timely treatment for recovery. So, the establishment of novel drugs is of utmost importance against the disease. The metabolites extracted from the cotton plant, *G. hirsutum*, obtained after structure-based drug discovery, were found to be the potential inhibitors of the adenosyl-L-homocysteine hydrolase (NfSAHH), an important protein to treat PAM*.* Not only did our top compounds show better binding affinity than the reference compound, but they also exhibited a similar interaction profile. The top ten compounds exhibited zero or one violation of Lipinski’s rule of five, while curcumin and piceid exhibited high GI absorption and favorable skin permeability. The top three metabolites, namely curcumin, heliocide H2, and piceid, show the ability to cross the blood-brain barrier and maintain relatively stable concentrations in the brain intracellular compartment. Overall, the RMSD plots showed that the heliocide H2 is the most promising candidate, while curcumin and piceid may need further optimization to improve their stability. Heliocide H2 stays within the binding pocket over the entire 100 ns simulation. Binding energy values, although decreased, remained negative, which means their interactions remain favorable. The obtained results suggest that the top hit metabolites have the potency to inhibit the growth and division of *Naegleria fowleri* by blocking its NfSAHH protein and should be subjected to wet lab experimental strategies for further investigations.

Future work will focus on further optimizing the top metabolites by introducing structural modifications to enhance their stability and binding affinity. Limitations of the study, such as reliance on computational predictions, will be addressed by integrating advanced modeling approaches and experimental techniques to improve accuracy and reliability. While this study focused on NfSAHH as the primary target, future investigations may broaden the scope to include other essential drug targets in *Naegleria fowleri* to develop a comprehensive therapeutic strategy.

## Supporting information

S1 Fig Molecular interaction analysis of Dotriacontanol (Blue) and Melissic acid (Purple): 2D schematic representation of Dotriacontanol (A) and Melissic acid (B) with hydrogen-bonded residue circles, binding of Dotriacontanol and Melissic acid in the active site of protein (green) (C), 3D interactions of Dotriacontanol (D) and Melissic acid (E) with active site residues (green) of protein. (TIF)

S2 Fig Molecular interaction analysis of stearic acid (purple) and 6,6'-dimethoxygossypol orange): 2D schematic representation of stearic acid (A) and 6,6'-dimethoxygossypol (B) with hydrogen-bonded residue circles, binding of stearic acid and 6,6'-dimethoxygossypol in the active site of protein (green) (C), and 3D interactions of stearic acid (D) and 6,6'-dimethoxygossypol (E) with active site residues (green) of protein. (TIF)

S3 Fig Molecular interaction analysis of Phytosphingosine 2 (yellow) and Methyl stearate (purple): 2D schematic representation of Phytosphingosine 2 (A) and Methyl stearate (B) with hydrogen-bonded residue circles, binding of Phytosphingosine 2 and Methyl stearate in the active site of protein (green) (C), 3D interactions of Phytosphingosine 2 (D) and Methyl stearate (E) with active site residues (green) of protein. (TIF)

S4 Fig Molecular interaction analysis of 6-methoxygossypol (sand): 2D schematic representation of 6-methoxygossypol (A) with hydrogen-bonded residue circles, binding of 6-methoxygossypol in the active site of protein (green) (B), 3D interactions of 6-methoxygossypol (C) with active site residues (green) of protein. (TIF)

S5 Fig 2D (A) and 3D (C) representations of the binding of the catalytic residues of protein at the important pharmacophoric domains of Curcumin and the key pharmacophoric features of Curcumin indicated by F1, F2, F3, and F4 (B). 2D (D) and 3D (F) representations of the binding of the catalytic residues of protein at the important pharmacophoric domains of Piceid and the key pharmacophoric features of Piceid indicated by F1, F2, F3, F4, and F5 (E). (TIF)

S1 Table Library of secondary metabolites of the cotton plant *Gossypium hirsutum* with their structures, nature, activities, location and the docking scores against the NfSAHH protein of a brain eating parasite *Naegleria fowleri.* (DOCX)

S2 Table Drug-likeness analysis of the top 50 phytochemicals of the Cotton Plant and the standard compound. (DOCX)

S3 Table Medicinal Chemistry of the top 10 metabolites and the standard compound by SwissADME server. (DOCX)

S4 Table Target fishing analysis of the top ten hits by Swiss target prediction server. (DOCX)

## References

[1] Tillery L, Barrett K, Goldstein J, Lassner JW, Osterhout B, Tran NL, et al. Naegleria fowleri: Protein structures to facilitate drug discovery for the deadly, pathogenic free-living amoeba. PLoS One. 2021;16(3):e0241738. doi: 10.1371/journal.pone.0241738 33760815 PMC7990177

[2] Marciano-Cabral F, Cabral GA. The immune response to Naegleria fowleri amebae and pathogenesis of infection. FEMS Immunol Med Microbiol. 2007;51(2):243–59. doi: 10.1111/j.1574-695X.2007.00332.x 17894804

[3] De Jonckheere JF. Molecular definition and the ubiquity of species in the genus Naegleria. Protist. 2004;155(1):89–103. doi: 10.1078/1434461000167 15144061

[4] Siddiqui R, Khan NA. Is ritual cleansing a missing link between fatal infection and brain-eating amoebae?. Clin Infect Dis. 2012;54(12):1817–8. doi: 10.1093/cid/cis309 22423138

[5] Shakoor S, Beg MA, Mahmood SF, Bandea R, Sriram R, Noman F, et al. Primary amebic meningoencephalitis caused by Naegleria fowleri, Karachi, Pakistan. Emerg Infect Dis. 2011;17(2):258–61. doi: 10.3201/eid1702.100442 21291600 PMC3204751

[6] Yoder JS, Straif-Bourgeois S, Roy SL, Moore TA, Visvesvara GS, Ratard RC, et al. Primary amebic meningoencephalitis deaths associated with sinus irrigation using contaminated tap water. Clin Infect Dis. 2012;55(9):e79-85. doi: 10.1093/cid/cis626 22919000 PMC11307261

[7] Major Robert H, Moser M. The New England Journal of Medicine Downloaded from nejm.org at UNIVERSITY OF OTAGO on November 13, 2015. For personal use only. No other uses without permission. From the NEJM Archive. Copyright © 2009 Massachusetts Medical Society. All rights reserved. 1956;9.

[8] Patras D, Andujar JJ. Meningoencephalitis due to Hartmannella (Acanthamoeba). Am J Clin Pathol. 1966;46(2):226–33. 5912486

[9] Callicott JH Jr, Nelson EC, Jones MM, dos Santos JG, Utz JP, Duma RJ, et al. Meningoencephalitis due to pathogenic free-living amoebae. Report of two cases. JAMA. 1968;206(3):579–82. doi: 10.1001/jama.1968.03150030035007 5695577

[10] Cerva L, Novăk K. Amoebic meningoencephalitis: 16 fatalities. Science. 1968;160(3823):92. doi: 10.1126/science.160.3823.92 5642317

[11] Schuster FL, Visvesvara GS. Opportunistic amoebae: challenges in prophylaxis and treatment. Drug Resist Updat. 2004;7(1):41–51. doi: 10.1016/j.drup.2004.01.002 15072770

[12] Lomas J, Anderson GM, Domnick-Pierre K, Vayda E, Enkin MW, Hannah WJ. Do practice guidelines guide practice? The effect of a consensus statement on the practice of physicians. N Engl J Med. 1989;321(19):1306–11. doi: 10.1056/NEJM198911093211906 2677732

[13] Petrovska BB. Historical review of medicinal plants’ usage. Pharmacogn Rev. 2012;6(11):1–5. doi: 10.4103/0973-7847.95849 22654398 PMC3358962

[14] Parasitic T, Unit D. Antiparasitic properties of medicinal plants and other naturally occurring products Parasitic. 2001.;50. 10.1016/s0065-308x(01)50032-9 11757332

[15] Ranasinghe S, Armson A, Lymbery AJ, Zahedi A, Ash A. Medicinal plants as a source of antiparasitics: an overview of experimental studies. Pathog Glob Health. 2023;117(6):535–53. doi: 10.1080/20477724.2023.2179454 36805662 PMC10392325

[16] Egbuta MA, McIntosh S, Waters DLE, Vancov T, Liu L. Biological importance of cotton by-products relative to chemical constituents of the cotton plant. Molecules. 2017;22(1):93. doi: 10.3390/molecules22010093 28067842 PMC6155835

[17] Pal D, Sahu P, Sethi G, Wallace CE, Bishayee A. Gossypol and its natural derivatives: multitargeted phytochemicals as potential drug candidates for oncologic diseases. Pharmaceutics. 2022;14(12):2624. doi: 10.3390/pharmaceutics14122624 36559116 PMC9787675

[18] Siddiqui R, Mungroo MR, Anuar TS, Alharbi AM, Alfahemi H, Elmoselhi AB, et al. Antiamoebic properties of laboratory and clinically used drugs against naegleria fowleri and balamuthia mandrillaris. Antibiotics (Basel). 2022;11(6):749. doi: 10.3390/antibiotics11060749 35740156 PMC9220410

[19] Tehlivets O, Malanovic N, Visram M, Pavkov-Keller T, Keller W. S-adenosyl-L-homocysteine hydrolase and methylation disorders: yeast as a model system. Biochim Biophys Acta. 2013;1832(1):204–15. doi: 10.1016/j.bbadis.2012.09.007 23017368 PMC3787734

[20] Thomford NE, Senthebane DA, Rowe A, Munro D, Seele P, Maroyi A, et al. Natural products for drug discovery in the 21st century: innovations for novel drug discovery. Int J Mol Sci. 2018;19(6):1578. doi: 10.3390/ijms19061578 29799486 PMC6032166

[21] Murgueitio MS, Bermudez M, Mortier J, Wolber G. In silico virtual screening approaches for anti-viral drug discovery. Drug Discov Today Technol. 2012;9(3):e219–25. doi: 10.1016/j.ddtec.2012.07.009 24990575 PMC7105918

[22] Dib H, Abu-Samha M, Younes K, Abdelfattah MAO. Evaluating the physicochemical properties-activity relationship and discovering new 1,2-dihydropyridine derivatives as promising inhibitors for pim1-kinase: evidence from principal component analysis, molecular docking, and molecular dynamics studies. Pharmaceuticals (Basel). 2024;17(7):880. doi: 10.3390/ph17070880 39065731 PMC11279803

[23] Daina A, Michielin O, Zoete V. SwissADME: a free web tool to evaluate pharmacokinetics, drug-likeness and medicinal chemistry friendliness of small molecules. Sci Rep. 2017;7 42717. doi: 10.1038/srep42717 28256516 PMC5335600

[24] Frechen S, Solodenko J, Wendl T, Dallmann A, Ince I, Lehr T, et al. A generic framework for the physiologically-based pharmacokinetic platform qualification of PK-Sim and its application to predicting cytochrome P450 3A4-mediated drug-drug interactions. CPT Pharmacometrics Syst Pharmacol. 2021;10(6):633–44. doi: 10.1002/psp4.12636 33946131 PMC8213412

[25] Willmann S, Thelen K, Lippert J. Integration of dissolution into physiologically-based pharmacokinetic models III: PK-Sim®. J Pharm Pharmacol. 2012;64(7):997–1007. doi: 10.1111/j.2042-7158.2012.01534.x 22686345

[26] Daina A, Michielin O, Zoete V. SwissTargetPrediction: updated data and new features for efficient prediction of protein targets of small molecules. Nucleic Acids Res. 2019;47(W1):W357–64. doi: 10.1093/nar/gkz382 31106366 PMC6602486

[27] Bowers KJ, Chow DE, Xu H, Dror RO, Eastwood MP, Gregersen BA, et al. Scalable algorithms for molecular dynamics simulations on commodity clusters. ACM/IEEE SC 2006 Conference (SC’06). 2006. doi: 10.1109/sc.2006.54

[28] Hildebrand PW, Rose AS, Tiemann JKS. Bringing molecular dynamics simulation data into view. Trends Biochem Sci. 2019;44(11):902–13. doi: 10.1016/j.tibs.2019.06.004 31301982

[29] Sastry GM, Adzhigirey M, Day T, Annabhimoju R, Sherman W. Protein and ligand preparation: parameters, protocols, and influence on virtual screening enrichments. J Comput Aided Mol Des. 2013;27(3):221–34. doi: 10.1007/s10822-013-9644-8 23579614

[30] Stewart S, Ivy MA, Anslyn EV. The use of principal component analysis and discriminant analysis in differential sensing routines. Chem Soc Rev. 2014;43(1):70–84. doi: 10.1039/c3cs60183h 23995750

[31] Yamin R, Ahmad I, Khalid H, Perveen A, Abbasi SW, Nishan U, et al. Identifying plant-derived antiviral alkaloids as dual inhibitors of SARS-CoV-2 main protease and spike glycoprotein through computational screening. Front Pharmacol. 2024;15 1369659. doi: 10.3389/fphar.2024.1369659 39086396 PMC11288853

[32] Kusakabe Y, Ishihara M, Umeda T, Kuroda D, Nakanishi M, Kitade Y, et al. Structural insights into the reaction mechanism of S-adenosyl-L-homocysteine hydrolase. Sci Rep. 2015;5 16641. doi: 10.1038/srep16641 26573329 PMC4647836

[33] Tillery L, Barrett K, Goldstein J, Lassner JW, Osterhout B, Tran NL, et al. Naegleria fowleri: Protein structures to facilitate drug discovery for the deadly, pathogenic free-living amoeba. PLoS One. 2021;16(3):e0241738. doi: 10.1371/journal.pone.0241738 33760815 PMC7990177

[34] Mukesh B, Rakesh K. ISSN 2229-3566 Review Article molecular docking: a review Bachwani Mukesh *, Kumar Rakesh. Int J Res Ayurveda Pharm. 2011;2:1746–51.

[35] Siddiqui R, Khan NA. Primary amoebic meningoencephalitis caused by Naegleria fowleri: an old enemy presenting new challenges. PLoS Negl Trop Dis. 2014;8(8):e3017. doi: 10.1371/journal.pntd.0003017 25121759 PMC4133175

[36] Repaci A, Ardizzoni A, Poluzzi E, Pagotto U. Ce Pt E Us Cr Ip T Pt Us Cr T. 2022:1–43. doi: DOIorIdentifier

[37] Lipinski CA. Lead- and drug-like compounds: the rule-of-five revolution. Drug Discov Today Technol. 2004;1(4):337–41. doi: 10.1016/j.ddtec.2004.11.007 24981612

[38] Ghose AK, Viswanadhan VN, Wendoloski JJ. A knowledge-based approach in designing combinatorial or medicinal chemistry libraries for drug discovery. 1. A qualitative and quantitative characterization of known drug databases. J Comb Chem. 1999;1(1):55–68. doi: 10.1021/cc9800071 10746014

[39] Li J, Holsworth D, Hu L. Molecular properties that influence the oral bioavailability of drug candidates. Chemtracts. n.d.;16 439–42.

[40] Egan WJ, Merz KM Jr, Baldwin JJ. Prediction of drug absorption using multivariate statistics. J Med Chem. 2000;43(21):3867–77. doi: 10.1021/jm000292e 11052792

[41] Ahmad I, Khalid H, Perveen A, Shehroz M, Nishan U, Rahman FU, et al. Identification of novel quinolone and quinazoline alkaloids as phosphodiesterase 10a inhibitors for parkinson’s disease through a computational approach. ACS Omega. 2024;9(14):16262–78. doi: 10.1021/acsomega.3c10351 38617664 PMC11007772

[42] Brenk R, Schipani A, James D, Krasowski A, Gilbert IH, Frearson J, et al. Lessons learnt from assembling screening libraries for drug discovery for neglected diseases. ChemMedChem. 2008;3(3):435–44. doi: 10.1002/cmdc.200700139 18064617 PMC2628535

[43] Daina A, Michielin O, Zoete V. SwissADME: a free web tool to evaluate pharmacokinetics, drug-likeness and medicinal chemistry friendliness of small molecules. Sci Rep. 2017;7 42717. doi: 10.1038/srep42717 28256516 PMC5335600

[44] Ertl P, Schuffenhauer A. Estimation of synthetic accessibility score of drug-like molecules based on molecular complexity and fragment contributions. J Cheminform. 2009;1(1):8. doi: 10.1186/1758-2946-1-8 20298526 PMC3225829

[45] Lin L, Wong H. Predicting Oral Drug Absorption: Mini Review on Physiologically-Based Pharmacokinetic Models. Pharmaceutics. 2017;9(4):41. doi: 10.3390/pharmaceutics9040041 28954416 PMC5750647

[46] Cacciatore I, Ciulla M, Marinelli L, Eusepi P, Di Stefano A. Advances in prodrug design for Parkinson’s disease. Expert Opin Drug Discov. 2018;13(4):295–305. doi: 10.1080/17460441.2018.1429400 29361853

[47] Konofagou EE. Optimization of the ultrasound-induced blood-brain barrier opening. Theranostics. 2012;2(12):1223–37. doi: 10.7150/thno.5576 23382778 PMC3563154

[48] Stalmans S, Bracke N, Wynendaele E, Gevaert B, Peremans K, Burvenich C, et al. Cell-penetrating peptides selectively cross the blood-brain barrier In Vivo. PLoS One. 2015;10(10):e0139652. doi: 10.1371/journal.pone.0139652 26465925 PMC4605843

[49] Poovaiah N, Davoudi Z, Peng H, Schlichtmann B, Mallapragada S, Narasimhan B, et al. Treatment of neurodegenerative disorders through the blood-brain barrier using nanocarriers. Nanoscale. 2018;10(36):16962–83. doi: 10.1039/c8nr04073g 30182106

[50] Hammarlund-Udenaes M, Bredberg U, Fridén M. Methodologies to assess brain drug delivery in lead optimization. Curr Top Med Chem. 2009;9(2):148–62. doi: 10.2174/156802609787521607 19200002

[51] Ambudkar SV, Kimchi-Sarfaty C, Sauna ZE, Gottesman MM. P-glycoprotein: from genomics to mechanism. Oncogene. 2003;22(47):7468–85. doi: 10.1038/sj.onc.1206948 14576852

[52] Zhuang X, Lu C. PBPK modeling and simulation in drug research and development. Acta Pharm Sin B. 2016;6(5):430–40. doi: 10.1016/j.apsb.2016.04.004 27909650 PMC5125732

[53] Jones HM, Gardner IB, Collard WT, Stanley PJ, Oxley P, Hosea NA, et al. Simulation of human intravenous and oral pharmacokinetics of 21 diverse compounds using physiologically based pharmacokinetic modelling. Clin Pharmacokinet. 2011;50(5):331–47. doi: 10.2165/11539680-000000000-00000 21456633

[54] Shah M, Khan F, Ahmad I, Deng C-L, Perveen A, Iqbal A, et al. Computer-aided identification of Mycobacterium tuberculosis resuscitation-promoting factor B (RpfB) inhibitors from Gymnema sylvestre natural products. Front Pharmacol. 2023;14 1325227. doi: 10.3389/fphar.2023.1325227 38094882 PMC10716330

[55] Galati S, Stefano MD, Martinelli E, Poli G, Tuccinardi T. Recent Advances in In Silico Target Fishing. 2021;1–18. 10.3390/molecules26175124 PMC8433825 34500568

[56] Shah M, Jaan S, Shehroz M, Sarfraz A, Asad K, Wara TU, et al. Deciphering the immunogenicity of monkeypox proteins for designing the potential mRNA Vaccine. ACS Omega. 2023;8(45):43341–55. doi: 10.1021/acsomega.3c07866 38024731 PMC10652822

[57] Hou T, Wang J, Li Y, Wang W. Assessing the performance of the MM/PBSA and MM/GBSA methods. 1. The accuracy of binding free energy calculations based on molecular dynamics simulations. J Chem Inf Model. 2011;51(1):69–82. doi: 10.1021/ci100275a 21117705 PMC3029230

[58] Grosso M, Kalstein A, Parisi G, Roitberg AE, Fernandez-Alberti S. On the analysis and comparison of conformer-specific essential dynamics upon ligand binding to a protein. J Chem Phys. 2015;142(24):245101. doi: 10.1063/1.4922925 26133456

