# Supplementary figures and images for: Exploring cotton plant compounds for novel treatments against brain-eating Naegleria fowleri: An In-silico approach
Source: PLoS One. 2025 Feb 24;20(2):e0319032. doi: 10.1371/journal.pone.0319032 (PMC11849825; doi:10.1371/journal.pone.0319032)

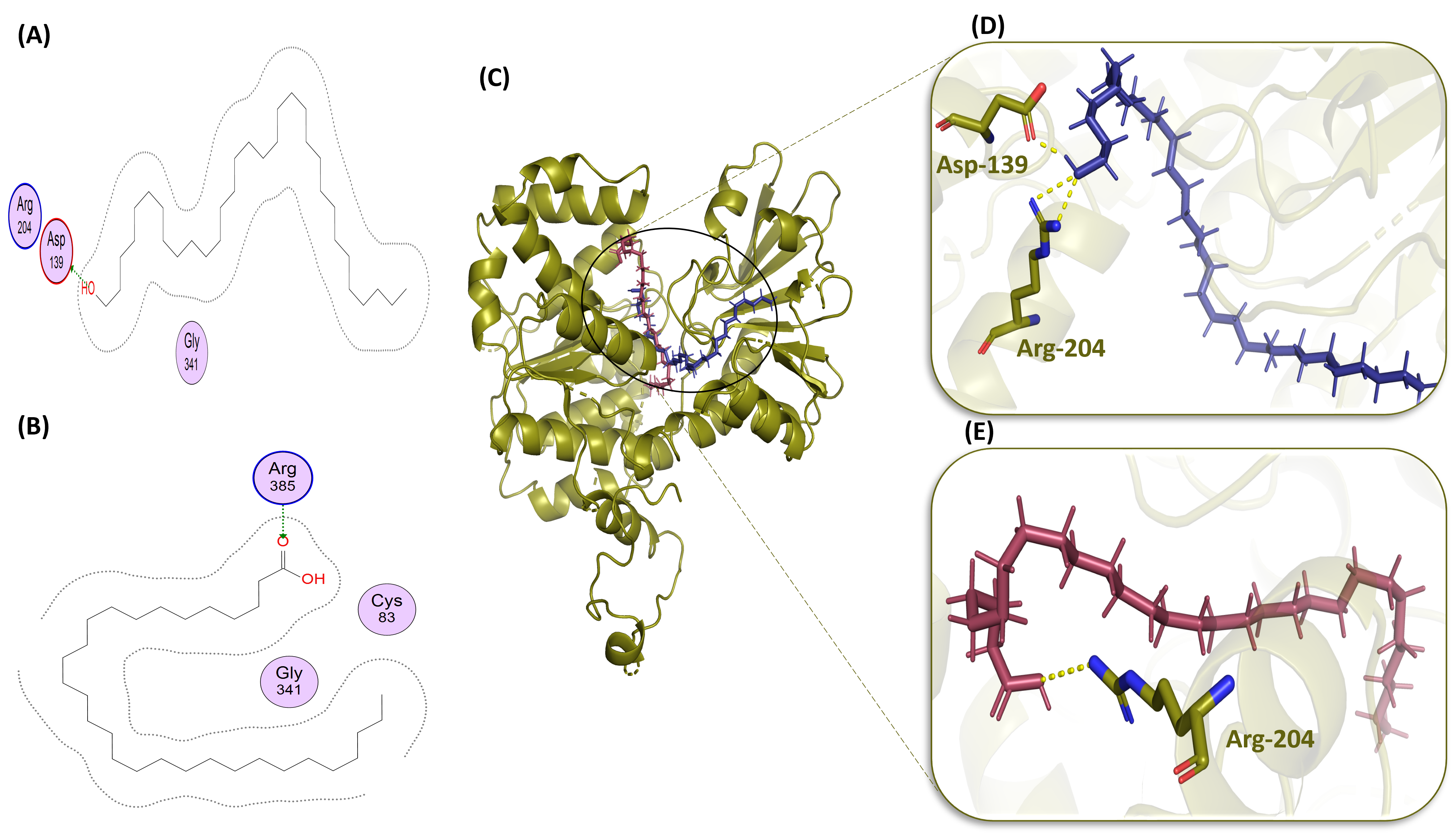

Supplement: S1 Fig — (TIF) [file pone.0319032.s001.tif]

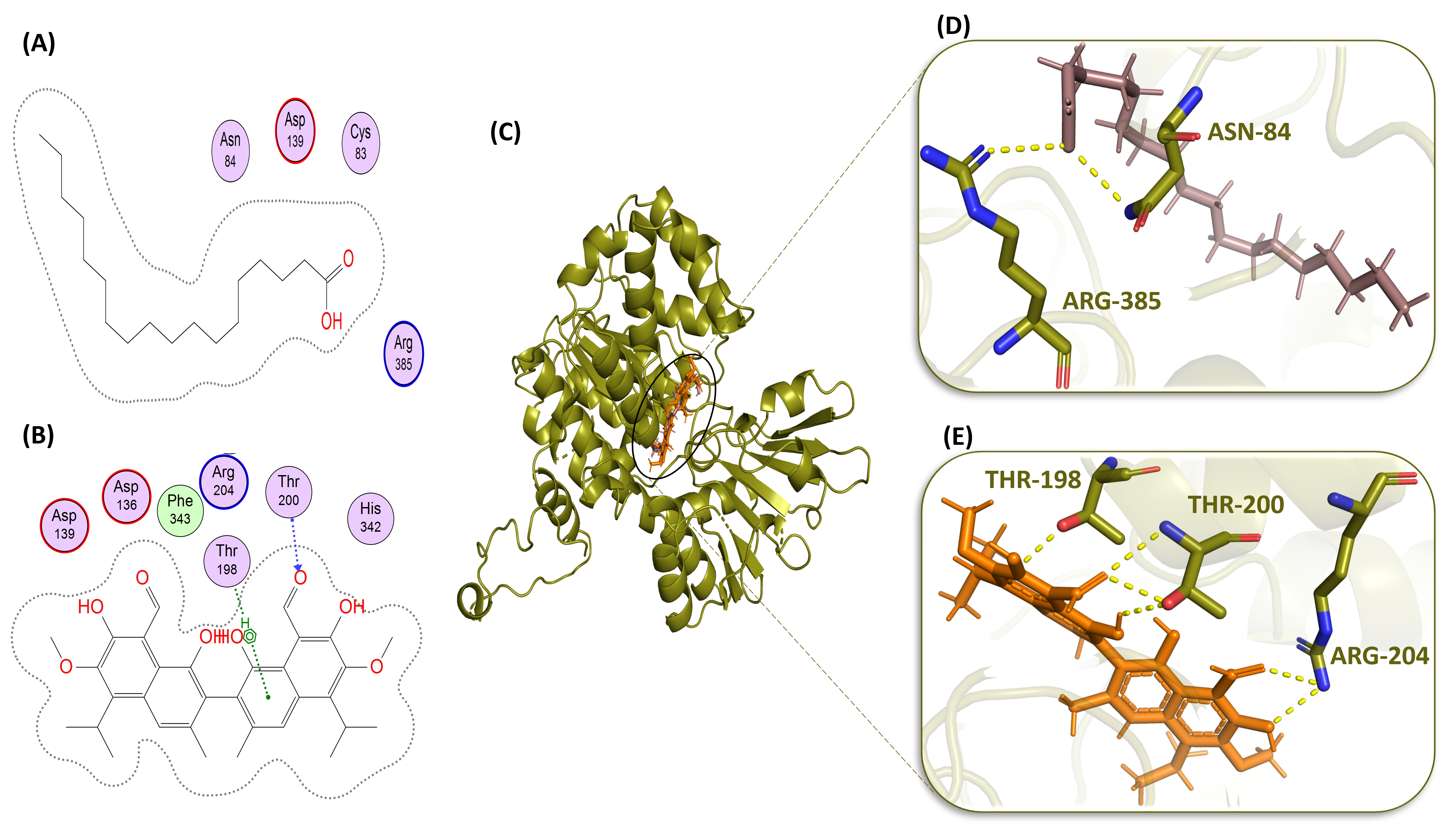

Supplement: S2 Fig — (TIF) [file pone.0319032.s002.tif]

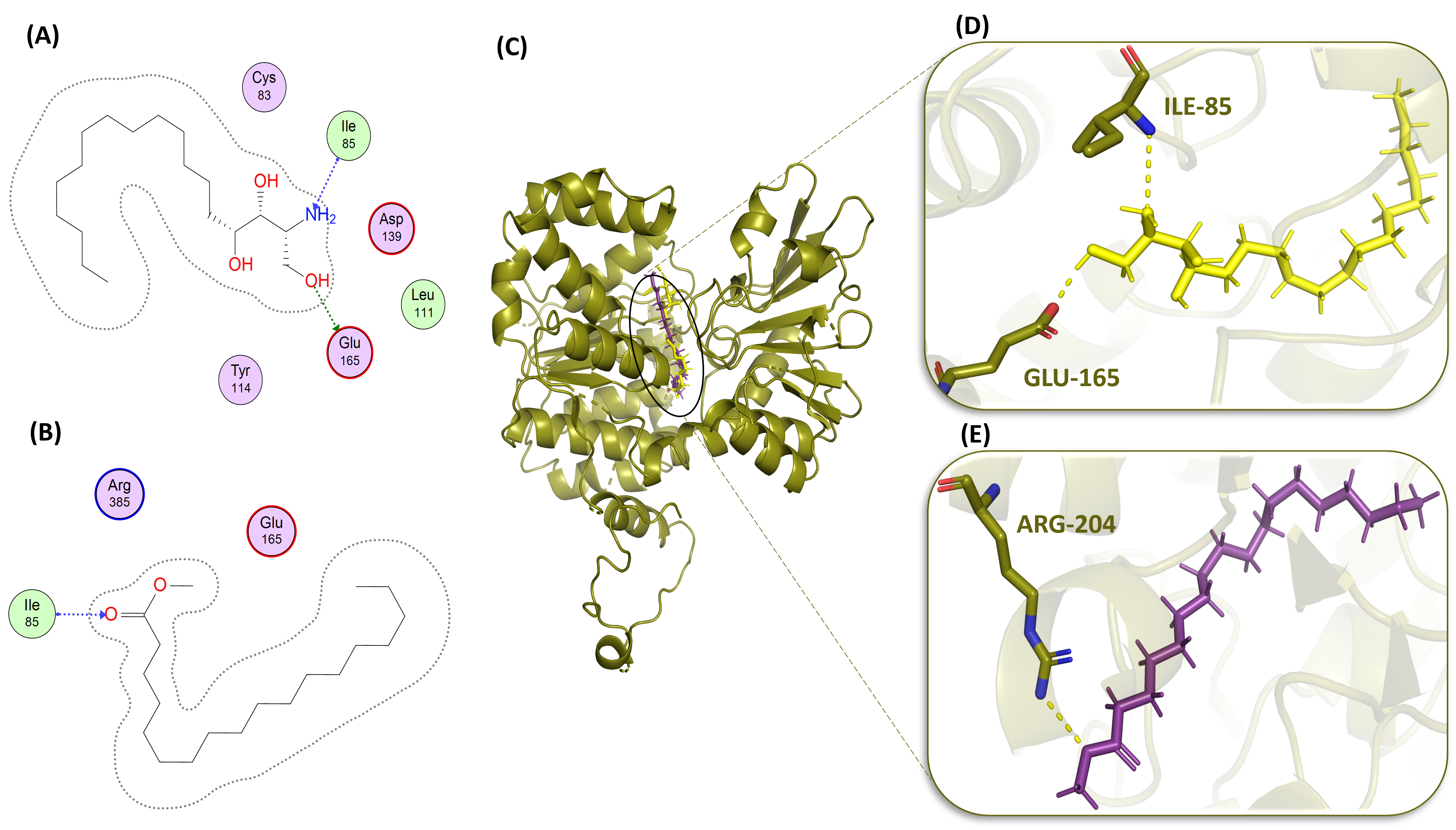

Supplement: S3 Fig — (TIF) [file pone.0319032.s003.tif]

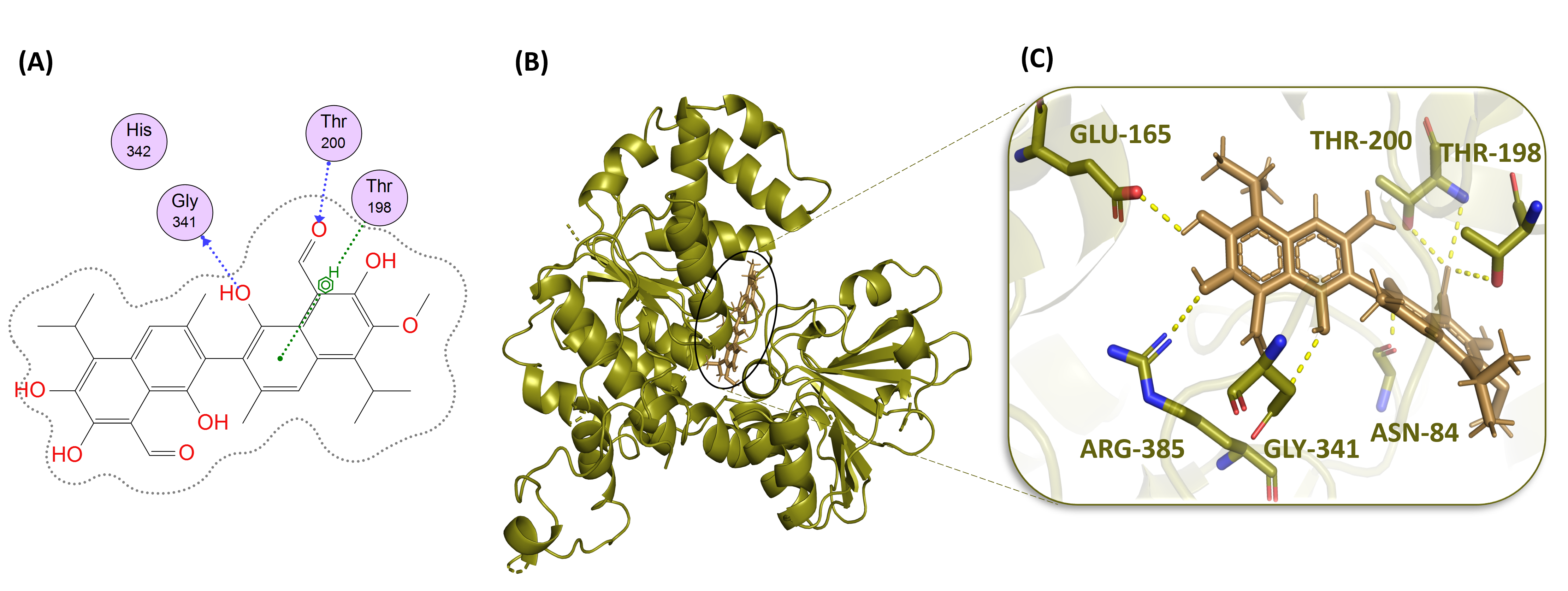

Supplement: S4 Fig — (TIF) [file pone.0319032.s004.tif]

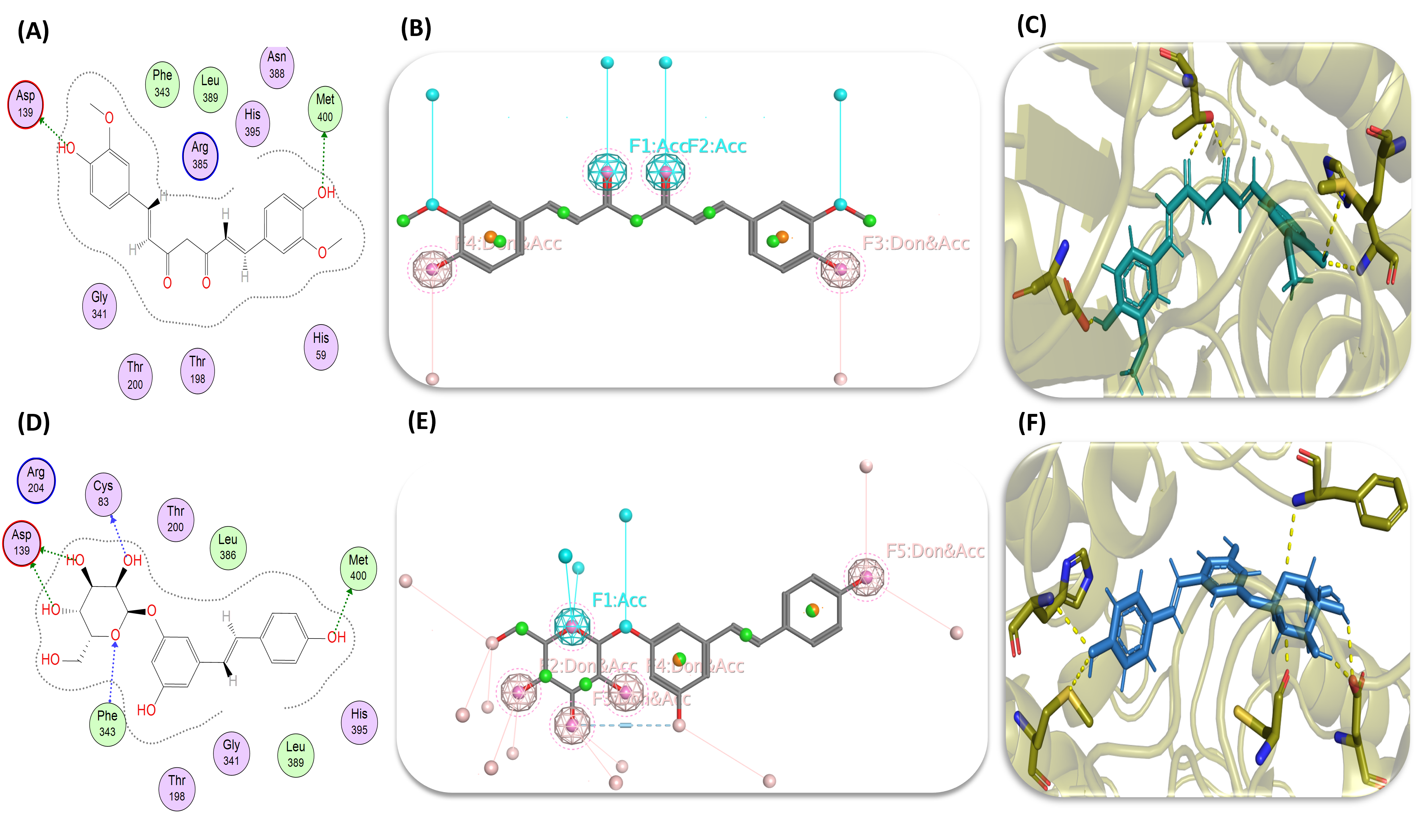

Supplement: S5 Fig — (TIF) [file pone.0319032.s005.tif]
