# Supplementary material for: Exploring cotton plant compounds for novel treatments against brain-eating Naegleria fowleri: An In-silico approach
Source: PLoS One. 2025 Feb 24;20(2):e0319032. doi: 10.1371/journal.pone.0319032 (PMC11849825; doi:10.1371/journal.pone.0319032)
Supplement: S1 Table — (DOCX) [file pone.0319032.s006.docx]

**S1 Table.** Library of secondary metabolites of the cotton plant *Gossypium hirsutum* with their structures, nature, activities, location and the docking scores against the NfSAHH protein of a brain eating parasite *Naegleria fowleri.*

| **Sr. No** | **Name** | **Structures** | **Type of chemical** | **Part of plant** | **Activities** | **References** | **Docking Scores** |
| --- | --- | --- | --- | --- | --- | --- | --- |
|  | Dotriacontanol |  | Alcohol | Leaves | Anti-inflammatory, anti-tumor and antidiabetic | [1] | -10.756 |
|  | Tamarixetin 7-glucoside |  | Flavonoid | Flower petals | Antioxidant and anticancer | [2] | -10.487 |
|  | Tiliroside |  | Flavonoid | Flowers | Anti-inflammatory and antioxidant | [3] | -10.328 |
|  | Quercetin-3-O-robinoside |  | Flavonoid | Seed | Antioxidant | [4] | -10.314 |
|  | Melissic acid (triacontanoic acid) |  | Saturated Fatty acid | Seeds and leaves | Antioxidant, antimicrobial, antitumour, antiviral, antiallergic, anti-inflammatory, and also flatulence inhibiting effects | [1] | -10.256 |
|  | Sexangularetin 3-glucoside-7-rhamnoside |  | Flavonoid | Immature flower buds |  | [5] | -9.882 |
|  | Quercetin 3-diglucoside |  | Flavonoid | Anthers and seed | Antioxidant | [6] | -9.803 |
|  | 13,15-dihydroxy -7-O- (6’-O -sulfate-b-D-glucopyranosyl)-  desoxyhemigossypol |  | Sulfated cadinenetype sesquiterpene | Seeds | Antiparasitic, anticancer, and antiviral activities | [7] | -9.613 |
|  | Squalene |  | Saturated fatty acid | Seeds | Emollient, antioxidant and antitumor | [8] | -9.588 |
|  | Nicotiflorin |  | Flavonoid | Flower petals, leaves, and seed | Anti-inflammatory, antioxidant, antibacterial, antiviral, analgesic, and neuroprotective | [9] | -9.583 |
|  | Curcumin |  | Curcuminoid | Leaves, roots, bolts | Powerful epigenic regulator in many diseases, such as neurological disorders, inflammatory diseases, diabetes, and cancers | [10] | -9.582 |
|  | 2,14-Epoxy-1,3,5,7,9-cadinapentaene-8,9,12-triol |  | Terpene derivative | Leaves, bolls, stalks, and stems |  | [7] | -9.552 |
|  | Quercetin-3'-glucoside |  | Flavonoid | Flowers, anthers | Antioxidant | [6] | -9.449 |
|  | 6,6′ -dimethoxygossypol |  | Terpenoid aldehyde | Roots | Anticancer | [11] | -9.416 |
|  | Quercimeritrin |  | Flavonoid | Flowers/petals |  | [9] | -9.246 |
|  | Phytosphingosine 2 |  | Sphingoid base | Leaves | Anti-inflammatory and antipsoriatic | [12] | -9.123 |
|  | Gossypitrin |  | Flavonoid | Flower petals, anthers | Antibacterial | [9] | -9.074 |
|  | Hirsutrin (iso quercetin) |  | Flavonoid | Flower and Leaves | Antioxidant, anticarcinogenic, antidiabetic and anti-allergic | [13] | -9.044 |
|  | Spiraeoside |  | Flavonoid | Flower petals, and seed | Antioxidant, anti-inflammatory and antitumor | [2] | -9.037 |
|  | Rutin |  | Flavonoid | Flower petals, anthers, leaves, hypocotyls, seed, and callus | Antibacterial, antiprotozoal, antitumor, anti-inflammatory, antiallergic, antiviral, antihypertensive, and cytoprotective | [9] | -8.968 |
|  | Quercetin 3-glycosides |  | Flavonoid | Flowers, leaves, barks and seeds | Anti-inflammatory, anti-lipoperoxidant, antitumoral, antimicrobial, anti-diabetic, anti-atherosclerotic, and cardioprotective activities | [14] | -8.882 |
|  | Quercitrin |  | Flavonoid | Leaves |  | [15] | -8.881 |
|  | Methyl stearate |  | Ester | Seeds | Antibacterial and antifungal | [8] | -8.881 |
|  | Stearic acid (octadecanoic acid) |  | Saturated Fatty acid | Seeds and leaves | Antibacterial | [1] | -8.867 |
|  | Piceid |  | Stilbene | Leaves | Cardioprotective, neuroprotective and antioxidant | [16] | -8.836 |
|  | Heliocide H2 |  | Terpene derivative | Leaves, bolls, stalks, and stems |  | [7] | -8.808 |
|  | 6-methoxygossypol |  | Terpenoid aldehyde | Roots | Anticancer | [11] | -8.731 |
|  | Prunin |  | Flavanone glycoside | Leaves | Antiviral | [12] | -8.725 |
|  | Hyperoside |  | Flavonoid | Flowers | Anticancer, anti-inflammatory, antibacterial, antiviral, antidepressant, and organ protective | [3] | -8.707 |
|  | Isoquercitrin |  | Flavonoid | Flowers, leaves, cotyledons, and seed | Antihypertensive, antiallergic, and antioxidant | [9] | -8.630 |
|  | Arachidic acid |  | Saturated fatty acid | Seeds | Protect against CNS injuries | [17] | -8.628 |
|  | Stigmast-5-en-3-ol, (3 beta) |  | Sterol | Seeds | Anxiolytic & sedative effects, analgesic, immunomodulatory, antimicrobial, anticancer, anti – inflammatory, lipid lowering effect, hepatoprotective, protective effect against NAFLD and respiratory diseases, wound healing effect, antioxidant and anti-diabetic activities | [8] | -8.593 |
|  | Phytol |  | Terpene derivative | Leaves, bolls, stalks, and stems |  | [1] | -8.496 |
|  | Linoleic acid (octadecadienoic acid) |  | Unsaturated fatty acid | Seeds | Anti-cancer, anti-oxidation, anti-atherosclerosis and improving immuno-responses | [1][18] | -8.496 |
|  | Trifolin |  | Flavonoid | Flower petals | Antifungal | [9] | -8.465 |
|  | Heliocide H1; 7-Me ether |  | Terpene derivative | Leaves, bolls, stalks, and stems |  | [19] | -8.438 |
|  | Palmitoleic acid (9-hexadecanoic acid) |  | Saturated Fatty acid | Seeds and leaves | Anti-inflammatory | [1] | -8.419 |
|  | Heliocide H3; 3-Me ether |  | Terpene derivative | Leaves, bolls, stalks, and stems |  | [19] | -8.380 |
|  | Heliocide H3 |  | Terpene derivative | Leaves, bolls, stalks, and stems | Insecticidal | [7] | -8.288 |
|  | Gossypin |  | Flavonoid | Flower petals, anthers | Antioxidant, anti-inflammatory, neuroprotective, anti-cancer, anti-tumor, and anti-diabetic | [9] | -8.271 |
|  | 11,14-Eicosadienoic acid |  | Unsaturated fatty acid | Leaves and seeds | Anti-inflammatory and hormonal activity | [1] | -8.253 |
|  | Gossypol |  | Phenolic acid analog | Seeds | Male antifertility activity, antiviral and anticancer activity | [20] | -8.242 |
|  | Elaidic acid (octadecenoic acid) |  | Unsaturated fatty acid | Seeds | Antioxidant, anti-diabetic, anticancer and apoptosis-inducing activities | [1][18] | -8.192 |
|  | Taxifolin |  | Flavanonol | Ovules, fiber, roots, cotyledons, and leaves | Anti-Alzheimer, antimicrobial, anticancer, hepatoprotective, cardioprotective, and antiangiogenic | [21] | -8.173 |
|  | Heliocide H2; 3-Me ether |  | Terpene derivative | Leaves, bolls, stalks, and stems |  | [19] | -8.127 |
|  | Eriodictyol |  | Flavanone | Ovules, fibre, roots, cotyledons, and leaves | Antioxidant, analgesic, antidiabetic, anti-inflammatory, cardioprotective, neuroprotective, and hepatoprotective | [21] | -8.101 |
|  | Astragalin |  | Flavonoid | Leaves | Anti-inflammatory and antioxidant | [16][9] | -8.094 |
|  | Naringenin |  | Flavanone | Hypocotyls, ovules, fibre, roots, cotyledons & leaves | Antioxidant, antitumor, antiviral, antibacterial, anti-inflammatory, antiadipogenic and cardioprotective | [21][22] | -8.079 |
|  | Neophytadiene |  | Sesquiterpenoid | Seeds | Anti-inflammatory and antimicrobial | [8] | -8.054 |
|  | Heptadecanoic acid |  | Saturated fatty acid | Leaves | Inhibits cell proliferation | [12] | -8.031 |
|  | Gossyrubilone |  | Phenolic acid analog | Seeds | Antimicrobial and anti-inflammatory | [19] | -8.026 |
|  | Chlorogenic acid |  | Phenol | Leaves, bolls, stalk and stems | Anti-oxidant and anti-mutagenic activity | [23][24] | -7.992 |
|  | Palmitic acid (hexadecanoic acid) |  | Saturated Fatty acid | Seeds and leaves | Regulators of inflammatory processes and various secretions and antibacterial activity | [1] | -7.951 |
|  | 3,3’,4’,5,7-pentahydroxyflavan; (2S,3R)-form |  | Phenol | Leaves, bolls, stalk and stems | Cytotoxic and phytotoxic activity | [25] | -7.928 |
|  | Catechin |  | Flavonoid | Leaves | Anti-microbial, antiviral, anti-inflammatory, anti-allergenic, and anti-cancer | [16][26] | -7.897 |
|  | Epicatechin |  | Flavonoid | Leaves | Cardiovascular and neuropsychological treatment | [16][26] | -7.893 |
|  | Herbacetin |  | Flavonoid | Flowers, leaves, barks and seeds | Antioxidant, anti-inflammatory and anticancer | [18] | -7.876 |
|  | Linolenic acid (octadecatrienoic acid) |  | Unsaturated fatty acid | Seeds | Anti-metabolic syndrome, anticancer, antiinflammatory, anti-oxidant, anti-obesity, neuroprotection, and regulation of the intestinal flora properties. | [1][18] | -7.829 |
|  | 13-hydroxy-7-O- (6’-O- sulfate-b-D- glucopyranosyl)- desoxyhemigossypol |  | Sulfated cadinenetype sesquiterpene | Seeds | Antiparasitic, anticancer,  and antiviral activities | [7] | -7.786 |
|  | Kaempferol |  | Flavonoid | Flowers, leaves, barks and seeds | Antioxidant, anti-inflammatory, antimicrobial, anticancer, cardioprotective, neuroprotective, antidiabetic, antiosteoporotic, estrogenic/antiestrogenic, anxiolytic, analgesic, and antiallergic activities | [18] | -7.777 |
|  | 3-p-coumaroylquinic acid |  | Phenolic compound | Leaves | Hypoglycemic activity | [16] | -7.662 |
|  | Leucodelphinidin |  | Leucoanthocyanidin | Anther, boll valves, stem bark, root bark | Antidiabetic | [26] | -7.659 |
|  | Proanthocyanidins |  | Condensed Tannin | Leaves | Antioxidant, antibacterial, antiviral, anti-inflammatory and anti-allergic activities | [23] | -7.654 |
|  | Strigol |  | Terpenoid | Roots | Germination stimulant | [27][28][29] | -7.626 |
|  | Malvidin |  | Anthocyanin | Leaves | Antioxidant and anti-inflammatory | [15] | -7.607 |
|  | Quercetin |  | Flavonoid | Flowers, leaves, barks and seeds | Antioxidant, anti-inflammatory, antibacterial, antiviral, radical-scavenging, gastroprotective, and immune-modulatory activities | [23] | -7.586 |
|  | Tamarixetin |  | Flavonoid | Flower petals | Antioxidant and anticancer | [30] | -7.579 |
|  | Octadecanol |  | Saturated fatty alcohol | Leaves | Anti-Parkinsonian properties, lipid metabolism regulation, relief of muscle fatigue, cardiovascular protection, liver protection, anti-inflammatory properties, epithelial cell protection | [12] | -7.575 |
|  | Heliocide H1 |  | Terpene derivative | Leaves, bolls, stalks, and stems |  | [7] | -7.573 |
|  | Myricetin |  | Flavonoid | Ovules and fibres from flower buds and bolls | Antioxidant, anticancer, antidiabetic and anti-inflammatory | [31] | -7.540 |
|  | Genistein |  | Isoflavone | Hypocotyls | Antioxidant, antiangiogenic, anthelmintic, and anticancer | [22] | -7.465 |
|  | Quercetin-3-O-neohesperidoside |  | Flavonoid | Seed | Anti-inflammatory | [4] | -7.463 |
|  | 2,3,8,9-Tetrahydroxy-1,3,5,7,9-cadinapentaen-14-al; 3-Me ether |  | Terpene derivative | Leaves, bolls, stalks, and stems |  | [32] | -7.424 |
|  | Leucocyanidin |  | Leucoanthocyanidin | Anther, boll valves, stem bark, root bark | Antioxidant, capillarotropic, cardioprotective and UV-protective | [26] | -7.415 |
|  | Hexadecanal |  | Aldehyde | Seeds | Affects human aggression | [8] | -7.394 |
|  | Aromadendrin |  | Flavanonol | Ovules, fiber, roots, cotyledons, and leaves | Regulate immune responses | [21] | -7.387 |
|  | Cyanidin |  | Anthocyanidin | Flower petals and leaves | Anti-inflammatory, anticancer, antidiabetic, antitoxic, cardiovascular and nervous protection | [9] | -7.322 |
|  | Gossypetin |  | Flavonoid | Leaves | Antioxidant, anti-atherosclerotic, and anticancer | [16] | -7.162 |
|  | Tricetin |  | Flavonoid | Leaves | Anticancer, antioxidant, antiviral and antihistaminic | [12] | -7.134 |
|  | 2,3,9-trihydroxy-1,3,5,7,9- cadinapentaen-14-al; 3-Me ether |  | Terpene | Leaves, stem, seeds and flower buds | Phytoalexin | [33] | -7.124 |
|  | Bisabolene |  | Sesquiterpene | Leaves | Anticonvulsant agent | [34] | -7.048 |
|  | Pterostilbene |  | Stilbene | Leaves | Anti-tumor, antioxidant, anti-inflammation, and neuroprotective | [16] | -7.040 |
|  | Hemigossypol |  | Sesquiterpenoid aldehydes | Roots | Antiparasitic, anticancer, antiapoptotic and antiviral activities | [11] | -7.014 |
|  | Myristic acid (tetradecanoic acid) |  | Saturated Fatty acid | Seeds and leaves | Antifungal, antiviral, anticancer and antiparasitic | [1] | -6.982 |
|  | Piceatannol |  | Stilbene | Leaves | Wound healing, anti-ageing, antioxidant and anti-acne | [16] | -6.981 |
|  | Gallocatechin |  | Flavanol | Hypocotyls/stem steles, anther, boll valves, stem bark, root bark | Antibacterial, antioxidant, antimalarial, antiulcer, and antiplasmodic | [26] | -6.960 |
|  | Kaempferide |  | Flavonoid | Flower petals | Antibacterial, antifungal and antiprotozoal | [35] | -6.934 |
|  | Epigallocatechin |  | Flavanol | Anther, boll valves, stem bark, root bark | Reduce inflammation, aid weight loss and prevent heart and brain diseases | [26] | -6.930 |
|  | 1,3,5,10-Bisabolatetraen-7-ol |  | Terpene derivative | Leaves, bolls, stalks, and stems |  | [36] | -6.893 |
|  | T*rans*-resveratrol |  | Stilbene | Seeds | Prevent vascular diseases, cancers, viral infections, and neurodegenerative processes | [37] | -6.884 |
|  | 2,8,9-trihydroxy-1,3,5,7,9- cadinapentaen-14-al; 8-deoxy |  | Terpene | Leaves, stem, seeds and flower buds | Antifungal activity | [38] | -6.795 |
|  | Methoxyhemigossypol |  | Sesquiterpenoid aldehydes | Roots | Antiparasitic, anticancer, and antiviral activities | [11] | -6.794 |
|  | 1,3,5,9-Cadinatetraene; 7αH-form, 3-Hydroxy |  | Terpene derivative | Leaves, bolls, stalks, and stems |  | [39] | -6.777 |
|  | Gossonorol |  | Sesquiterpene | Leaves | Antibacterial and antifungal | [40] | -6.749 |
|  | 3,10-Dihydroxy-1,3,5,7-cadinatetraen-9-one |  | Terpene derivative | Leaves, bolls, stalks, and stems |  | [41] | -6.675 |
|  | 1,3,5,7,9-Cadinapentaene-3,9-diol |  | Terpene derivative | Leaves, bolls, stalks, and stems |  | [42] | -6.669 |
|  | Para-hemigossypolone |  | Sesquiterpenoid Aldehyde Quinone | Flower buds | Antibacterial | [43] | -6.664 |
|  | Syringic acid |  | Phenolic acid | Seeds | Anti-inflammatory, anti-mitogenic, anti-oxidant, anti-cancer, anti-diabetic and hepatoprotective | [24] | -6.650 |
|  | β-sitosterol |  | Triterpenes | Leaves | Antimicrobial activity, anti-hypercholesteraemic and anti-inflammatory activity | [1] | -6.642 |
|  | β-bisabolol |  | Sesquiterpenoid | Flowers | Aromatic properties, anti-inflammatory effects, anti-carcinogenic activity, anti-microbial and anti-oxidative properties | [44][36][45] | -6.596 |
|  | Protocatechiuc acid |  | Phenolic Acid | Seeds | Anti-oxidative, anti-aging, anti-inflammatory, and nephroprotective activity | [23] | -6.572 |
|  | Heliocide H4; 3-Me ether |  | Terpene derivative | Leaves, bolls, stalks, and stems |  | [19] | -6.562 |
|  | Farnesene |  | Sesquiterpene | Leaves | Anti-oxidant effects | [34] | -6.557 |
|  | Aurones |  | Flavonoid | Flowers, leaves, barks and seeds | Antiviral, antibacterial, antifungal, anti-inflammatory, antitumor, antimalarial, antioxidant, neuropharmacological activities | [46] | -6.545 |
|  | 1,3,5-Cadinatriene-3,9-diol; (7a,9a,10a)-form, 9-Ketone |  | Terpene derivative | Leaves, bolls, stalks, and stems |  | [47] | -6.469 |
|  | Uridine 1 |  | Nucleoside | Leaves | Manage neuropsychiatric deficits associated with cerebrovascular diseases | [12] | -6.403 |
|  | 1,3,5-Cadinatriene-3,9-diol; (7ß,10a)-form, 9-Ketone |  | Terpene derivative | Leaves, bolls, stalks, and stems |  | [47] | -6.345 |
|  | Ferulic acid |  | Phenolic acid | Seeds | Antioxidant, antiinflammatory, antimicrobial, antiallergic, hepatoprotective, anticarcinogenic, antithrombotic | [24] | -6.318 |
|  | Isopropyl-β-D-thiogalactopyranoside |  | S-glycosyl compound | Leaves | Induce protein expression | [12] | -6.311 |
|  | 1,3,5,7,9-Cadinapentaene-3,9-diol; 3-Me ether |  | Terpene derivative | Leaves, bolls, stalks, and stems |  | [42] | -6.293 |
|  | Cadinene |  | Monoterpene | Leaves and calyx | Antimicrobial and antifungal | [34] | -6.265 |
|  | 1,3,5-Cadinatriene-3,9,10-triol; (7ß,9ß,10a)-form, 9-O-ß-D-Glucopyranoside |  | Terpene derivative | Leaves, bolls, stalks, and stems |  | [48] | -6.187 |
|  | 2,14-Epoxy-1,3,5,7,9-cadinapentaene-8,9-diol |  | Terpene derivative | Leaves, bolls, stalks, and stems |  | [38] | -6.181 |
|  | Hydroxycinnamic acid (p-coumaric acid) |  | Phenolic acid | Seeds | Treatment of cancer, diabetes, lungs, cardiovascular diseases and also have antimicrobial and anti-inflammatory activities | [23][24] | -6.132 |
|  | Copaene |  | Sesquiterpene | Leaves | Antioxidant | [34] | -6.104 |
|  | Guaiene |  | Monoterpene | Leaves and calyx | Antimicrobial, antioxidant, analgesic, anti-inflammatory, antiplatelet, antithrombotic, aphrodisiac, antidepressant, antimutagenic, antiemetic, fibrinolytic and cytotoxic activities | [34] | -6.023 |
|  | Heliocide H4 |  | Terpene derivative | Leaves, bolls, stalks, and stems | Insecticidal | [7] | -6.012 |
|  | Bergamotene |  | Sesquiterpene | Leaves | Anti-cancer activity | [34] | -6.011 |
|  | β-amyrin |  | Triterpenes | Leaves | Anti-oxidant | [1] | -5.906 |
|  | Caryophyllene oxide |  | Epoxide | Seeds and leaves | Cytotoxic activity, phytogrowth inhibition, analgesic and anti-inflammatory activity | [36][49][50] | -5.842 |
|  | Gallic acid |  | Phenolic Acid | Seeds | Antioxidant activity, cytotoxic activity | [24] | -5.808 |
|  | Humulene |  | Sesquiterpene | Leaves | Anti-inflammatory properties, aromatic properties and cytotoxic activity | [34] | -5.746 |
|  | Caffeic acid |  | Phenolic compound | Seeds | Antioxidant, anti-inflammatory and antiproliferative | [51] | -5.745 |
|  | Caryophyllene |  | Sesquiterpene | Leaves | Cytotoxic activity, phytogrowth inhibition, analgesic and anti-inflammatory activity | [34][52][36] | -5.723 |
|  | 5-Methoxytryptamine 2 |  | Aromatic ether | Leaves | Closely related to the neurotransmitters, serotonin and melatonin. | [12] | -5.702 |
|  | Synephrine 1 |  | p-synephrine | Leaves | Sympathomimetic | [12] | -5.617 |
|  | Scopoletin |  | Coumarin | Leaves | Anti-spasmodic and anti-inflammatory activity | [53] | -5.569 |
|  | Sorbitol |  | Sugar alcohol | Leaves | Laxative | [12] | -5.537 |
|  | Ocimene |  | Terpene | Leaves | Defensive responses to herbivory | [54][34] | -5.459 |
|  | Myrcene |  | Monoterpene | Leaves and flowers | Analgesic effects, anti-microbial activity, anti-inflammatory activity, anti-catabolic activity | [54][55] | -5.459 |
|  | Gossypurpurin |  | Phenolic acid analog | Seeds | Antimicrobial and anti-inflammatory | [56] | -5.433 |
|  | Xylitol |  | Alcohol | Leaves | Promote better dental health | [12] | -5.289 |
|  | Alpha terpinene |  | Terpene | Flower buds | Insecticidal | [55] | -5.253 |
|  | Alpha-phellandrene |  | Monoterpene | Leaves and calyx | Antitumoral, antinociceptive, larvicidal and insecticidal activities | [55] | -5.158 |
|  | *p*-Hydroxybenzoic acid |  | Phenolic Acid | Seeds | Antibacterial, anticancer, antidiabetic, antiaging, antiviral, and anti-inflammatory activities | [24] | -5.138 |
|  | Spathulenol |  | Sesquiterpene alcohol | Calyx | Antioxidant, anti-inflammatory, antiproliferative and antimycobacterial activities | [40] | -5.114 |
|  | 3(15),6-Caryophylladien-12-ol; (6E)-form |  | Terpene derivative | Leaves, bolls, stalks, and stems |  | [57] | -5.109 |
|  | Terpinolene |  | Monoterpene | Leaves and calyx | Anti-inflammatory | [55] | -5.092 |
|  | Camphene |  | Monoterpene | Leaves and flowers | Aromatic properties, antioxidants effects | [58][55] | -5.070 |
|  | Beta-phellandrene |  | Monoterpene | Leaves and calyx | Anti-microbial, insecticidal, anti-inflammatory, anti-cancer, wound healing, analgesic, and neuronal responses | [55] | -5.043 |
|  | Shikimic acid |  | Cyclohexanecarboxylic acid | Leaves | Antibacterial | [12] | -5.024 |
|  | 5-hyrdoxymethylfurfural (HMF) |  | Furfural | Leaves | Anti-oxidant,  Antimicrobial and Genotoxic | [12] | -5.008 |
|  | Gamma terpinene |  | Terpene | Flower buds | Antibacterial | [55] | -4.939 |
|  | Sabinene |  | Monoteroenoid | Flowers, leaves, barks and seeds | Anti-microbial activity, anti-oxidant activity | [58][54] | -4.920 |
|  | Benzoic acid |  | Phenolic acid | Seeds | Anti-microbial | [24] | -4.889 |
|  | Gentisic acid |  | Phenolic acid | Seeds | Antioxidant, anticarcinogenic, hepatoprotective, antimicrobial, analgesic, neuroprotective, and cardioprotective | [24] | -4.846 |
|  | Phthalic acid |  | Carboxylic acid | Seeds | Antimicrobial, allelopathic and insecticidal | [8] | -4.839 |
|  | α-thujene |  | Monoterpenoid | Flowers, leaves, barks and seeds | Pungent activity | [58] | -4.830 |
|  | Limonene |  | Monoterpene | Leaves and flowers | Flavouring properties. gastro-protective effects, anti-cancer and anti-inflammatory activity | [54][34][55] | -4.815 |
|  | Succinic acid |  | Carboxylic acid | Leaves | Antibiotic and curative agent | [12] | -4.760 |
|  | 3(15),6-Caryophylladien-12-ol; (6E)-form, 6a,7ß-Epoxide, Ac |  | Terpene derivative | Leaves, bolls, stalks, and stems |  | [57] | -4.681 |
|  | α-pinene |  | Monoterpene | Leaves and flowers | Gastro-protective effects, anti- microbial and ant-inflammatory effects | [54][55] | -4.675 |
|  | Threonic acid |  | Sugar acid | Leaves | Antioxidant | [12] | -4.652 |
|  | Norleucine 2 |  | Amino acid | Leaves | Antiviral | [12] | -4.512 |
|  | L-Malic acid |  | Carboxylic acid | Leaves | Used to improve the absorption of drugs and is used in amino acid infusions for the treatment of liver dysfunction or high blood ammonia | [12] | -4.418 |
|  | Furfuryl alcohol |  | Furfural | Leaves | Anti-oxidant | [12] | -4.396 |
|  | β-pinene |  | Monoterpene | Leaves and flowers | Gastro-protective effects, anti- microbial and ant-inflammatory effects | [54][55] | -4.378 |
|  | Erythritol |  | Polyol | Leaves | Organic insecticide | [12] | -4.357 |
|  | Glycine |  | Amino acid | Leaves | Antioxidant, anti-inflammatory, cryoprotective, and immunomodulatory in peripheral and nervous tissues | [12] | -4.032 |
|  | Glycolic acid |  | Carboxylic acid | Leaves | Antibacterial | [12] | -3.942 |
|  | Lactic acid |  | Carboxylic acid | Leaves | Anti pathogenic | [12] | -3.760 |
|  | Chrysanthemin |  | Anthocyanin | Flower/buds, leaves, cotyledons, anther tissue culture, boll valves, and stem bark | Antioxidant and anti-inflammatory | [59] | Inactive |
|  | Hydroxylamine |  | Amine | Leaves | Antioxidant | [12] | Inactive |
