# Supplementary material for: Exploring cotton plant compounds for novel treatments against brain-eating Naegleria fowleri: An In-silico approach
Source: PLoS One. 2025 Feb 24;20(2):e0319032. doi: 10.1371/journal.pone.0319032 (PMC11849825; doi:10.1371/journal.pone.0319032)
Supplement: S2 Table — (DOCX) [file pone.0319032.s007.docx]

**S2 Table.** Drug-likeness analysis of the top 50 phytochemicals of the Cotton Plant and the standard compound.

| **Sr. No** | **Compound** | **M. Formula** | **M. weight (g/mol)** | **HA** | **HBA** | **HBD** | **Log P** | **Log S** | **MR** | **TPSA**  **(Å²)** | **Lipinski** |
| --- | --- | --- | --- | --- | --- | --- | --- | --- | --- | --- | --- |
|  | Neplanocin A  (standard) | C11H13N5O3 | 263.25 | 19 | 6 | 4 | 1.09 | -0.44 | 65.92 | 130.31 | Yes;  0 violation |
|  | Dotriacontanol | C32H66O | 466.87 | 33 | 1 | 1 | 8.10 | -10.70 | 157.10 | 20.23 | Yes;  1 violation:  MLOGP>4.15 |
|  | Tamarixetin 7-glucoside | C22H22O12 | 478.40 | 34 | 12 | 7 | 2.73 | -3.86 | 114.63 | 199.51 | No;  2 violations:  NorO>10, NHorOH>5 |
|  | Tiliroside | C30H26O13 | 594.52 | 43 | 13 | 7 | 2.99 | -4.93 | 149.51 | 216.58 | No;  3 violations:  MW>500, NorO>10, NHorOH>5 |
|  | Quercetin-3-O-robinoside | C33H40O21 | 772.66 | 54 | 21 | 13 | 0.86 | -2.91 | 173.50 | 348.58 | No;  3 violations:  MW>500, NorO>10, NHorOH>5 |
|  | Melissic acid (triacontanoic acid) | C30H60O2 | 452.80 | 32 | 2 | 1 | 7.08 | -10.07 | 148.10 | 37.30 | Yes;  1 violation:  MLOGP>4.15 |
|  | Sexangularetin 3-glucoside-7-rhamnoside | C28H32O16 | 624.54 | 44 | 16 | 9 | 2.21 | -3.13 | 145.59 | 258.43 | No;  3 violations:  MW>500, NorO>10, NHorOH>5 |
|  | Quercetin 3-diglucoside | C27H30O17 | 626.52 | 44 | 17 | 11 | 0.90 | -2.41 | 142.54 | 289.66 | No;  3 violations:  MW>500, NorO>10, NHorOH>5 |
|  | 13,15-dihydroxy -7-O- (6’-O -sulfate-b-D-glucopyranosyl)-  Desoxyhemigossypol | C22H28O12S | 516.52 | 35 | 12 | 7 | 2.28 | -2.02 | 120.70 | 211.82 | No;  3 violations:  MW>500, NorO>10, NHorOH>5 |
|  | Squalene | C30H50 | 410.72 | 30 | 0 | 0 | 2.28 | -2.02 | 143.48 | 0.00 | No;  3 violations:  MW>500, NorO>10, NHorOH>5 |
|  | Nicotiflorin | C27H30O15 | 594.52 | 42 | 15 | 9 | 2.79 | -3.42 | 139.36 | 249.20 | No;  3 violations:  MW>500, NorO>10, NHorOH>5 |
|  | Curcumin | C21H20O6 | 368.38 | 27 | 6 | 2 | 3.27 | -3.94 | 102.80 | 93.06 | Yes;  0 violation |
|  | 2,14-Epoxy-1,3,5,7,9-cadinapentaene-8,9,12-triol | C21H26O13S | 518.49 | 35 | 13 | 7 | 0.47 | -1.66 | 116.98 | 221.05 | No;  3 violations:  MW>500, NorO>10, NHorOH>5 |
|  | Quercetin-3'-glucoside | C21H20O12 | 464.38 | 33 | 12 | 8 | 2.02 | -3.04 | 110.16 | 210.51 | No;  2 violations:  NorO>10, NHorOH>5 |
|  | 6,6′-dimethoxygossypol | C32H34O8 | 546.61 | 40 | 8 | 4 | 4.23 | -7.91 | 157.83 | 133.52 | Yes;  1 violation:  MW>500 |
|  | Quercimeritrin | C21H20O12 | 464.38 | 33 | 12 | 8 | 1.54 | -3.04 | 110.16 | 210.51 | No;  2 violations:  NorO>10, NHorOH>5 |
|  | Phytosphingosine 2 | C18H39NO3 | 317.51 | 22 | 4 | 4 | 4.23 | -3.68 | 94.83 | 86.71 | Yes;  0 violation |
|  | Gossypitrin | C21H20O13 | 480.38 | 34 | 13 | 9 | 1.55 | -2.91 | 112.18 | 230.74 | No;  2 violations:  NorO>10, NHorOH>5 |
|  | Hirsutrin (iso quercetin) | C21H20O12 | 464.38 | 33 | 12 | 8 | 2.11 | -3.04 | 110.16 | 210.51 | No;  2 violations:  NorO>10, NHorOH>5 |
|  | Spiraeoside | C21H20O12 | 464.38 | 33 | 12 | 8 | 1.45 | -3.64 | 110.16 | 210.51 | No;  2 violations:  NorO>10, NHorOH>5 |
|  | Rutin | C27H30O16 | 610.52 | 43 | 16 | 10 | 1.58 | -3.30 | 141.38 | 269.43 | No;  3 violations:  MW>500, NorO>10, NHorOH>5 |
|  | Quercetin 3-glycosides | C27H30O17 | 626.52 | 44 | 17 | 11 | 0.90 | -2.41 | 142.54 | 289.66 | No;  3 violations:  MW>500, NorO>10, NHorOH>5 |
|  | Methyl stearate | C19H38O2 | 298.50 | 21 | 2 | 0 | 4.81 | -5.83 | 94.73 | 26.30 | Yes;  1 violation:  MLOGP>4.15 |
|  | Quercitrin | C21H20O11 | 448.38 | 32 | 11 | 7 | 1.27 | -3.33 | 109.00 | 190.28 | No;  2 violations:  NorO>10, NHorOH>5 |
|  | Stearic acid (octadecanoic acid) | C18H36O2 | 284.48 | 20 | 16 | 2 | 4.30 | -5.73 | 90.41 | 37.30 | Yes;  1 violation:  MLOGP>4.15 |
|  | Piceid | C20H22O8 | 390.38 | 28 | 8 | 6 | 1.75 | -2.90 | 100.00 | 139.84 | Yes;  1 violation:  NHorOH>5 |
|  | Heliocide H2 | C25H30O5 | 410.50 | 30 | 5 | 2 | 3.53 | -5.29 | 118.29 | 91.67 | Yes;  0 violation |
|  | 6-methoxygossypol | C31H32O8 | 532.58 | 39 | 8 | 5 | 3.07 | -7.69 | 153.36 | 144.52 | Yes;  1 violation:  MW>500 |
|  | Prunin | C21H22O10 | 434.39 | 31 | 10 | 6 | 2.38 | -2.97 | 103.69 | 166.14 | Yes;  1 violation:  NHorOH>5 |
|  | Hyperoside | C21H20O12 | 464.38 | 33 | 12 | 8 | 2.11 | -3.04 | 110.16 | 210.51 | No;  2 violations:  NorO>10, NHorOH>5 |
|  | Isoquercitrin | C21H20O12 | 464.38 | 33 | 12 | 8 | 2.11 | -3.04 | 110.16 | 210.51 | No;  2 violations:  NorO>10, NHorOH>5 |
|  | Arachidic acid | C20H40O2 | 312.53 | 22 | 2 | 1 | 4.56 | -6.44 | 100.03 | 37.30 | Yes;  1 violation:  MLOGP>4.15 |
|  | Stigmast-5-en-3-ol, (3 beta) | C47H84O2 | 681.17 | 49 | 2 | 0 | 9.02 | -14.02 | 219.88 | 26.30 | No;  2 violations:  MW>500, MLOGP>4.15 |
|  | Linoleic acid (octadecadienoic acid) | C18H32O2 | 280.45 | 20 | 2 | 1 | 4.14 | -5.05 | 89.46 | 37.30 | Yes;  1 violation:  MLOGP>4.15 |
|  | Phytol | C20H40O | 296.53 | 21 | 1 | 1 | 4.66 | -5.98 | 98.94 | 20.23 | Yes;  1 violation:  MLOGP>4.15 |
|  | Trifolin | C21H20O11 | 448.38 | 32 | 11 | 7 | 0.53 | -3.18 | 108.13 | 190.28 | No;  2 violations:  NorO>10, NHorOH>5 |
|  | Heliocide H1; 7-Me ether | C26H32O5 | 424.53 | 31 | 5 | 1 | 3.69 | -5.51 | 122.75 | 80.67 | Yes;  0 violation |
|  | Palmitoleic acid (9-hexadecanoic acid) | C16H30O2 | 254.41 | 18 | 2 | 1 | 3.53 | -4.70 | 80.32 | 37.30 | Yes;  0 violation |
|  | Heliocide H3; 3-Me ether | C26H32O6 | 440.53 | 32 | 6 | 2 | 3.65 | -5.26 | 124.22 | 100.90 | Yes;  0 violation |
|  | Heliocide H3 | C25H30O5 | 410.50 | 30 | 5 | 2 | 3.62 | -5.29 | 118.29 | 91.67 | Yes;  0 violation |
|  | Gossypin | C21H20O13 | 480.38 | 34 | 13 | 9 | 1.02 | -2.91 | 112.18 | 230.74 | No;  2 violations:  NorO>10, NHorOH>5 |
|  | 11,14-Eicosadienoic acid | C20H36O2 | 308.50 | 22 | 2 | 1 | 4.41 | -5.76 | 99.08 | 37.30 | Yes;  1 violation:  MLOGP>4.15 |
|  | Gossypol | C30H30O8 | 518.55 | 38 | 8 | 6 | 3.36 | -7.48 | 148.89 | 155.52 | No;  2 violations:  MW>500, NHorOH>5 |
|  | Elaidic acid (octadecenoic acid) | C18H34O2 | 282.46 | 20 | 2 | 1 | 4.27 | -5.41 | 89.94 | 37.30 | Yes;  1 violation:  MLOGP>4.15 |
|  | Taxifolin | C15H12O7 | 304.25 | 22 | 7 | 5 | 1.30 | -2.66 | 74.76 | 127.45 | Yes;  0 violation |
|  | Heliocide H2; 3-Me ether | C26H32O6 | 440.53 | 32 | 6 | 2 | 3.60 | -5.26 | 124.22 | 100.90 | Yes;  0 violation |
|  | Eriodictyol | C15H12O6 | 288.25 | 21 | 6 | 4 | 1.62 | -3.26 | 73.59 | 107.22 | Yes;  0 violation |
|  | Astragalin | C21H20O11 | 448.38 | 32 | 11 | 7 | 0.53 | -3.18 | 108.13 | 190.28 | No;  2 violations:  NorO>10, NHorOH>5 |
|  | Naringenin | C15H12O5 | 272.25 | 20 | 5 | 3 | 1.75 | -3.49 | 71.57 | 86.99 | Yes;  0 violation |
|  | Neophytadiene | C20H38 | 278.52 | 20 | 0 | 0 | 5.05 | -6.77 | 97.31 | 0.00 | Yes;  1 violation:  MLOGP>4.15 |
|  | Heptadecanoic acid | C17H34O2 | 270.45 | 19 | 2 | 1 | 4.11 | -5.37 | 85.60 | 37.30 | Yes;  1 violation:  MLOGP>4.15 |
