# Supplementary material for: Exploring cotton plant compounds for novel treatments against brain-eating Naegleria fowleri: An In-silico approach
Source: PLoS One. 2025 Feb 24;20(2):e0319032. doi: 10.1371/journal.pone.0319032 (PMC11849825; doi:10.1371/journal.pone.0319032)
Supplement: S3 Table — (DOCX) [file pone.0319032.s008.docx]

**S3 Table.** Medicinal Chemistry of the top 10 metabolites and the standard compound by SwissADME server.

| **Sr. No.** | **Compound** | **PAINS** | **Brenk** | **Lead-likeness** | **Synthetic accessibility** |
| --- | --- | --- | --- | --- | --- |
|  | **Neplanocin A**  **(standard)** | 0 alert | 1 alert: isolated_alkene | Yes | 3.87 |
|  | **Dotriacontanol** | 0 alert | 0 alert | No; 3 violations: MW>350, Rotors>7, XLOGP3>3.5 | 4.22 |
|  | **Melissic acid (triacontanoic acid)** | 0 alert | 0 alert | No; 3 violations: MW>350, Rotors>7, XLOGP3>3.5 | 3.98 |
|  | **Curcumin** | 0 alert | 2 alerts: beta_keto_anhydride, michael_acceptor_1 | No; 2 violations: MW>350, Rotors>7 | 2.97 |
|  | **6,6′ -dimethoxygossypol** | 0 alert | 1 alert: aldehyde | No; 2 violations: MW>350, XLOGP3>3.5 | 3.81 |
|  | **Phytosphingosine 2** | 0 alert | 0 alert | No; 2 violations: Rotors>7, XLOGP3>3.5 | 4.01 |
|  | **Methyl stearate** | 0 alert | 0 alert | No; 2 violations: Rotors>7, XLOGP3>3.5 | 2.76 |
|  | **Stearic acid (octadecanoic acid)** | 0 alert | 0 alert | No; 2 violations: Rotors>7, XLOGP3>3.5 | 2.54 |
|  | **Piceid** | 0 alert | 1 alert: stilbene | No; 1 violation: MW>350 | 4.82 |
|  | **Heliocide H2** | 2 alerts: catechol_A, keto_keto_gamma | 3 alerts: aldehyde, catechol, isolated_alkene | No; 2 violations: MW>350, XLOGP3>3.5 | 4.38 |
|  | **6-methoxygossypol** | 1 alert: catechol_A | 2 alerts: aldehyde, catechol | No; 2 violations: MW>350, XLOGP3>3.5 | 3.68 |
