# Supplementary material for: Exploring cotton plant compounds for novel treatments against brain-eating Naegleria fowleri: An In-silico approach
Source: PLoS One. 2025 Feb 24;20(2):e0319032. doi: 10.1371/journal.pone.0319032 (PMC11849825; doi:10.1371/journal.pone.0319032)
Supplement: S4 Table — (DOCX) [file pone.0319032.s009.docx]

**S4 Table.** Target fishing analysis of the top ten hits by Swiss target prediction server.

| **Sr. No.** | **Predicted targets** | **Probability** |
| --- | --- | --- |
| 1. | **Neplanocin A (standard)** |  |
|  | **-** |  |
| 2. | **Dotriacontanol** |  |
|  | Transient receptor potential cation channel subfamily M member 8 | 0.1928 |
|  | Carbonic anhydrase II | 0.1928 |
|  | Carbonic anhydrase I | 0.1928 |
|  | Carbonic anhydrase IV | 0.1928 |
|  | Androgen Receptor | 0.11739 |
| 3. | **Melissic acid (triacontanoic acid)** |  |
|  | Fatty acid binding protein adipocyte | 0.430164 |
|  | Peroxisome proliferator-activated receptor alpha | 0.430164 |
|  | Fatty acid binding protein muscle | 0.430164 |
|  | Fatty acid binding protein epidermal | 0.430164 |
|  | Peroxisome proliferator-activated receptor delta | 0.430164 |
| 4. | **Curcumin** |  |
|  | Monoamine oxidase A | 1 |
|  | Beta amyloid A4 protein | 1 |
|  | Histone acetyltransferase p300 | 1 |
|  | Prostaglandin E synthase | 1 |
|  | Toll-like receptor (TLR7/TLR9) | 1 |
| 5. | **6,6′ -dimethoxygossypol** |  |
|  | Aldose reductase | 0.666327 |
|  | Apoptosis regulator Bcl-X | 0.188005 |
|  | Transitional endoplasmic reticulum ATPase | 0.105306 |
|  | DNA-3-methyladenine glycosylase | 0.105306 |
|  | L-lactate dehydrogenase A chain | 0.096101 |
| 6. | **Phytosphingosine 2** |  |
|  | Sphingosine kinase 1 | 0.182601 |
|  | Alpha-L-fucosidase I | 0.135202 |
|  | Maltase-glucoamylase | 0.119404 |
|  | Sucrase-isomaltase | 0.119404 |
|  | Lysosomal alpha-glucosidase | 0.111502 |
| 7. | **Methyl stearate** |  |
|  | Carbonic anhydrase II | 0.237885 |
|  | Carbonic anhydrase I | 0.213126 |
|  | Fatty acid binding protein adipocyte | 0.097875 |
|  | Fatty acid binding protein muscle | 0.097875 |
|  | Fatty acid binding protein epidermal | 0.097875 |
| 8. | **Stearic acid (octadecanoic acid)** |  |
|  | Peroxisome proliferator-activated receptor alpha | 0.9293 |
|  | Peroxisome proliferator-activated receptor delta | 0.9293 |
|  | Fatty acid binding protein adipocyte | 0.71485 |
|  | Fatty acid binding protein epidermal | 0.71485 |
|  | Fatty acid binding protein muscle | 0.526361 |
| 9. | **Piceid** |  |
|  | Aldose reductase (by homology) | 0.123937 |
|  | Tyrosinase | 0.115737 |
|  | Adenosine A2a receptor (by homology) | 0.115737 |
|  | Sodium/glucose cotransporter 1 | 0.115737 |
|  | Cyclooxygenase-1 | 0.115737 |
| 10. | **Heliocide H2** |  |
|  | Maternal embryonic leucine zipper kinase | 0.106166 |
|  | Endoplasmin | 0.106166 |
|  | Heat shock protein HSP 90-alpha | 0.106166 |
|  | Bcl2-antagonist of cell death (BAD) | 0.106166 |
|  | Pyruvate dehydrogenase kinase isoform 1 | 0.106166 |
| 11. | **6-methoxygossypol** |  |
|  | Aldose reductase | 0.515968 |
|  | Apoptosis regulator Bcl-X | 0.148169 |
|  | Induced myeloid leukemia cell differentiation protein Mcl-1 | 0.130646 |
|  | Apoptosis regulator Bcl-2 | 0.130646 |
|  | L-lactate dehydrogenase A chain | 0.130646 |
